# Proteo-Trancriptomic Analyses Reveal a Large Expansion of Metalloprotease-Like Proteins in Atypical Venom Vesicles of the Wasp *Meteorus pulchricornis* (Braconidae)

**DOI:** 10.3390/toxins13070502

**Published:** 2021-07-19

**Authors:** Jean-Luc Gatti, Maya Belghazi, Fabrice Legeai, Marc Ravallec, Marie Frayssinet, Stéphanie Robin, Djibril Aboubakar-Souna, Ramasamy Srinivasan, Manuele Tamò, Marylène Poirié, Anne-Nathalie Volkoff

**Affiliations:** 1 INRAE, CNRS, ISA, Université Côte d’Azur, 06903 Sophia Antipolis, France; marylene.poirie@univ-cotedazur.fr; 2 CNRS, INP, Institution NeuroPhysiopathol, Aix-Marseille University, CEDEX, 13385 Marseille, France; maya.belghazi@univ-amu.fr; 3 IGEPP, INRAE, Institut Agro, Université de Rennes, 35653 Le Rheu, France; fabrice.legeai@inrae.fr (F.L.); stephanie.robin@inrae.fr (S.R.); 4 DGIMI, INRAE, University of Montpellier, 34095 Montpellier, France; marc.ravallec@inrae.fr (M.R.); marie.frayssinet@inrae.fr (M.F.); djibrilsa@gmail.com (D.A.-S.); 5 International Institute of Tropical Agriculture, Benin Research Station (IITA-Benin), Cotonou 08 BP 0932, Benin; M.Tamo@cgiar.org; 6 World Vegetable Center (WorldVeg), Shanhua, Tainan 74151, Taiwan; srini.ramasamy@worldveg.org

**Keywords:** *Meteorus pulchricornis*, parasitoid wasp, Braconidae, venomics, virus-like particles (VLPs), proteomic, transcriptomic, metalloproteases, DUF-4803 proteins

## Abstract

*Meteorus pulchricornis* (Ichneumonoidea, Braconidae) is an endoparasitoid wasp of lepidopteran caterpillars. Its parasitic success relies on vesicles (named *M. pulchricornis* Virus-Like Particles or MpVLPs) that are synthesized in the venom gland and injected into the parasitoid host along with the venom during oviposition. In order to define the content and understand the biogenesis of these atypical vesicles, we performed a transcriptome analysis of the venom gland and a proteomic analysis of the venom and purified MpVLPs. About half of the MpVLPs and soluble venom proteins identified were unknown and no similarity with any known viral sequence was found. However, MpVLPs contained a large number of proteins labelled as metalloproteinases while the most abundant protein family in the soluble venom was that of proteins containing the Domain of Unknown Function DUF-4803. The high number of these proteins identified suggests that a large expansion of these two protein families occurred in *M. pulchricornis*. Therefore, although the exact mechanism of MpVLPs formation remains to be elucidated, these vesicles appear to be “metalloproteinase bombs” that may have several physiological roles in the host including modifying the functions of its immune cells. The role of DUF4803 proteins, also present in the venom of other braconids, remains to be clarified.

## 1. Introduction

*Meteorus pulchricornis* (Ichneumonoidea, Braconidae, Euphorinae) is an endoparasitoid wasp that develops in the larval stages of a large number of lepidopteran species including several pests such as *Helicoverpa armigera* [1,2]. *M. pulchricornis* is widely distributed in Europe and thelytokous strains can be found in Japan and New-Zealand [2,3]. *M. pulchricornis* lays eggs in host caterpillars that continue to develop after parasitism (koinobiont lifestyle). To ensure successful parasitism, koinobiont endoparasitoids such as *M. pulchricornis* rely on various strategies to modulate the host internal physiological conditions in order to make them more suitable to the development of their eggs and larvae. These strategies comprise the production of factors in the female genital tract or by specialized embryonic cells released in the parasitized insect, the teratocytes [4]. Female-derived factors include proteins or vesicles made in the venom gland or in specialized ovarian tissues [5,6,7].

Virus-like particles produced in the calyx of female ovaries have been extensively studied in ichneumonid and braconid wasps. In these species, the particles are produced by viral machineries imbedded in wasp chromosomes as a result of virus genome integration events that occurred during wasp evolution [8,9,10,11,12,13]. These parasitoid ovarian particles contain either DNA molecules or proteins, and they originate from independent integration events. The best known are those from the polydnavirus (PDV) family. PDV particles enclose circular dsDNA molecules carrying wasp genes that are expressed in the cells of different tissues of the parasitized host. The products of these genes ensure successful parasitism by suppressing host immune responses and/or altering host larval development. 

Unlike other braconid endoparasitoid species, *M. pulchricornis* females do not produce viral particles in their ovaries. However, electron microscopy studies have indicated that the lumen of their venom glands and their reservoir are filled with numerous vesicles resembling virus particles, which have been named MpVLPs [14,15]. MpVLPs are produced in the cells of the venom gland and then stored in the venom reservoir. Injection of purified MpVLPs in *Pseudaletia separata* (*Mythimna separata*) host larvae strongly decreased the ability of host hemocytes to encapsulate fluorescent latex beads, and one specific type of hemocytes, granulocytes, showed rapid cytoskeleton change followed by apoptosis [14,15,16]. Therefore, MpVLPs appear to play an important role in parasitoid success by modulating the host immune response and protecting the parasitoid egg from the host encapsulation response.

MpVLPs have been described as single-membrane vesicles approximately 150 nm in diameter filled with electron-dense material that does not contain nucleic acids [15]. This feature makes them more similar to virus-like particles produced in the ovaries of the ichneumonid *Venturia canescens* [10] than to PDVs from other Braconids. Interestingly, up to now, and with the exception of *Meteorus* spp., only parasitoid species in the family Figitidae (mainly *Leptopilina* and *Ganaspis* species) have been reported to form vesicles in their venom gland [17,18]. Figitidae vesicles are also uniquely filled with proteins that target specialized host immune cells [19,20]. However, and in contrast to our current knowledge on VLPs/PDVs produced in wasp ovaries, a clear demonstration of the origin (cellular or viral derived) and exact mechanism of biogenesis of these wasp venom vesicles remains to be obtained [19,21,22,23].

To gain further insights on the nature and origin of *M. pulchricornis* venom and MpVLPs, we investigated in the present study the transcriptome of the venom gland and the proteome of the venom and purified MpVLPs. The aim was to complement a prior published study on the venom gland transcriptome that was based only on sequencing of a low number of clones (473 clones) from a conventional cDNA library of venom gland filaments [24]. In this previous study, many of the sequences were related to cellular components or unknown. Of these, several were selected to be knockdown by RNA interference and two, which were immunolocalized with MpVLPs, slightly affected the adhesion and spreading of host plasmatocytes suggesting these proteins may be involved in host immune suppression. However, single and double KO of these genes did not affect the success of wasp parasitism.

Here, Illumina sequencing of the *M. pulchricornis* venom gland transcriptome yielded nearly 17,000 predicted coding sequences (CDS). This database was used for proteomic analysis of the total venom extracted from the venom reservoir and MpVLPs purified from this venom. Among the most abundant proteins found in the venom and composing the MpVLPs, we identified a high number of Zn-metalloproteases and proteins containing the Domain-of-Unknown-Function-4803 (DUF4803) suggesting that a large expansion of these two protein families had occurred in *M. pulchricornis* lineage. Interestingly, since MpVLPs affect host immune cells and wasp metalloproteases have been implicated in impairing host cell immune functions, it is tempting to suggest a role for MpVLPs metalloproteases in this process. We did not find genes from known viruses abundantly expressed in the transcriptome of the *M. pulchricornis* venom gland, nor did we find viral proteins in the proteomic analyses of venom and MpVLPs. Although not definitive, these data suggest that MpVLPs do not have a viral origin in contrast to the ovarian calyx particles described in other braconids. 

## 2. Results and Discussion

### 2.1. M. pulchricornis Venom Gland and MpVLPs Secretion

**Figure 1:**
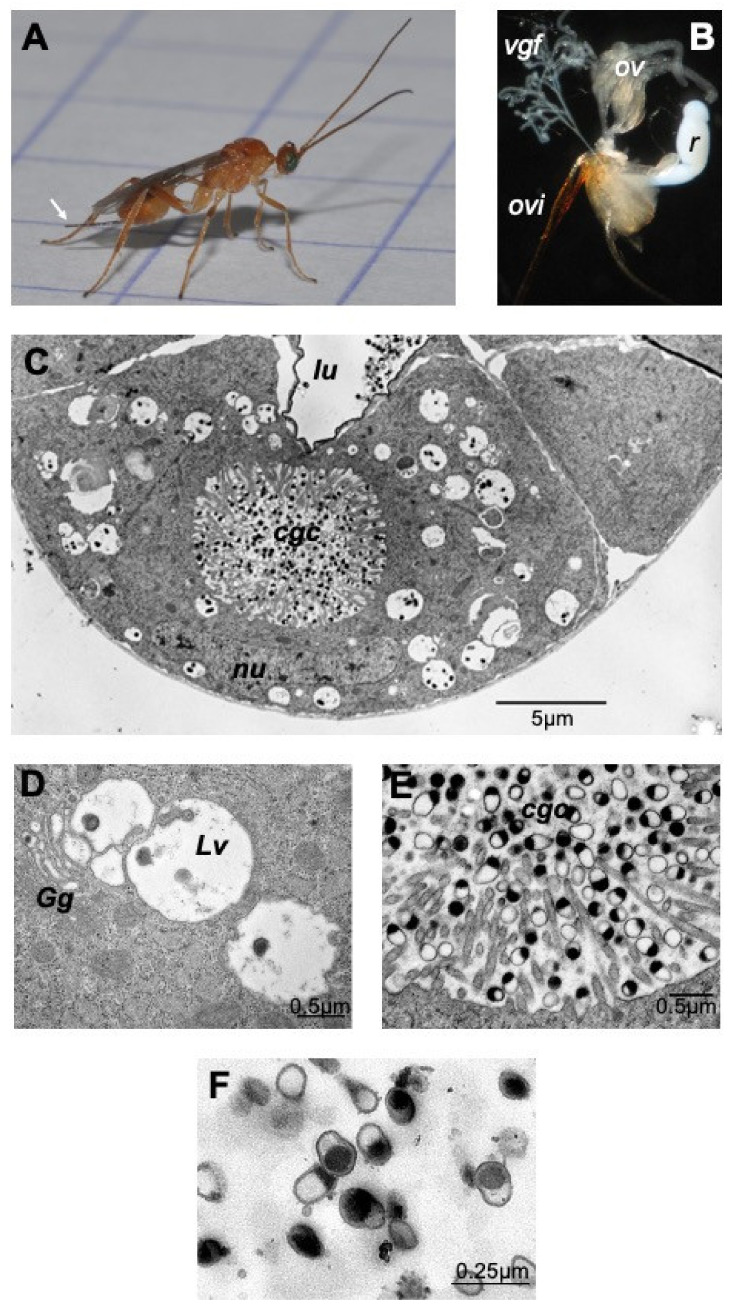
The venom apparatus of *Meteorus pulchricornis*. From the female wasp (**A**) the venom apparatus (**B**) was obtained by pulling out the ovipositor (ovi; white arrow in A). The ovarioles (ov), the two filamentous venom glands (*vgf*) and the large unique milky venom reservoir (*r*) are clearly visible. (**C**) TEM observation of a cross section of a venom gland filament showing a glandular cell with the secretory cell glandular canal (*cgc*) at its center that ends in the lumen of the collecting gland duct (*lu*). The nucleus (*nu*) surrounded by its nuclear envelope had and apparent normal shape. (**D**) MpVLPs may derive from the small electron dense vesicles contained within large cytoplasmic vesicles (*Lv*) found associated with the Golgi apparatus (*Gg*). (**E**) The internal canal (*cgc*) of the secretory gland cell, lined by microvilli, is filled with secreted mature MpVLPs. (**F**) MpVLPs purified by centrifugation of venom collected from *M. pulchricornis* venom reservoir (MpVLPs average longer diameter 250 nm, *n* = 20). These vesicles were apparently single membraned vesicles (membrane mean width 30 ± 13 nm, *n* = 17).

The dissected venom apparatus from *M. pulchricornis* female wasps (Figure 1A) consists of two filamentous venom glands and a large milky reservoir (Figure 1B). Observed by transmission electron microscopy (TEM), the cells of the gland (Figure 1C) show the classic type of glandular venom cells with an internal secretory cell canal (cell glandular canal, cgc) surrounded by microvilli [25]. The glandular cells showed a normal nucleus and had a cytoplasm filled with large vesicles, very often in close proximity to the Golgi apparatus (Figure 1D). The center of the cell canal in this species was packed with vesicles filled or half filled with an electron dense material, the MpVLPs (Figure 1E). These secreted vesicles were further observed in the lumen of the collecting duct running at the center of the gland where the cell duct opens (Figure 1C). These large vesicles enclosed small membranous vesicles filled with dark material, which may represent the precursor of secreted MpVLPs. MpVLPs purified by centrifugation (Figure 1F) from the venom in the reservoir (Figure 1B) have the same appearance as those observed in the secretory cell canal and the gland collecting duct, indicating that the purification procedure did not induce significant changes.

### 2.2. Analysis of M. pulchricornis Venom Gland Transcriptome

**Figure 2:**
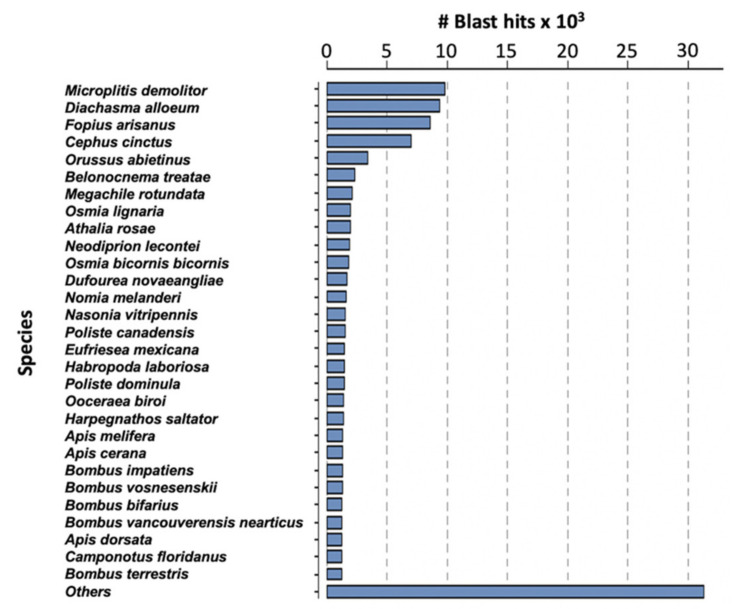
Species classified by the number of hits reported for *M. pulchricornis* CDS protein sequences against the NCBI NR database.

Assembly of the filamentous venom gland transcriptome yielded 14,926 contigs corresponding to 16,828 predicted coding sequences (CDS) (Table S1). The difference can be explained by the existence of chimeric contigs containing two or more tandem reading frames or with forward and reverse phase sequences for instance. The average length of the predicted CDS was 855 nucleotides (median 555 nt). Analyses were made on the 16,828 predicted proteins and, after BlastP similarity searches against the NCBI NR database, a total of 10,096 of these proteins matched a known protein. The highest number of matches was obtained with *Microplitis demolitor*, followed by *Diaschama alloeum* and *Fopius arisanus* which belong to the same braconid family as *M. pulchricornis* (Figure 2). 

To estimate the extent to which the *M. pulchricornis* transcriptomic dataset included orthologs from other related or distantly related parasitoid species (Figure S1A), orthology relationships were searched by Orthofinder using as input the venom CDSs of *M. pulchricornis* and predicted proteins from a series of available parasitoid genomes (the braconids *Aphidius ervi* Haliday, *Cotesia congregate* Say, *Diachasma alloeum*, *Fopius arisanus, Lysiphlebus fabarum* Marshall and *Microplitis demolitor* Wilkinson, the ichneumonids *Campoletis sonorensis* Cameron, *Hyposoter didymator* Thunberg and *Venturia canescens* Gravenhorst, the pteromalid *Nasonia vitripennis* Walker, plus *Apis mellifera* L. and *Drosophila melanogaster* Meigen as non-parasitoid species) and braconid venom gland transcriptomes (*Liragathis javana* Bhat & Gupta, *Psyttalia concolor* Szepligeti, *Bracon nigricans* Szepligeti) (see Table S2). This analysis showed that among the 16,828 predicted *M. pulchricornis* proteins, a total of 11,767 CDSs (70%) were distributed into 8315 orthogroups composed of 2 to 471 members while 5061 CDSs could not be assigned to an orthogroup (see Table S1). 

Of the 8315 orthogroups identified by Orthofinder, 122 contained only *M. puchricornis* proteins (from two to 99 sequences/orthogroups). The most abundant was OG000306 (99 proteins), which included proteins with similarities with metalloproteases or with the previously described hypothetical BAL70303.1 protein from *M. pulchricornis*, followed by OG0005090 (34 proteins) and OG0006398 (30 proteins), which corresponded to uncharacterized proteins. The majority of the orthogroups thus included sequences from other species, although in variable number. Orthogroups with a large majority of *M. pulchricornis* proteins included OG0000843 (67 of 68 entries were *M. pulchricornis* sequences), OG0001872 (50 of 51 entries) or OG0005678 (30 of 32 entries). All three also included sequences displaying similarities to metalloproteases or the BAL70303.1 protein (like OG000306 above). Note that the richest orthogroups (for instance OG0000003 (471 entries) or OG0000005 (327 entries)) were mainly composed of cellular proteins with orthologs from almost all analyzed species.

Then, to obtain functional labels, the 16,828 proteins were aligned against NCBI NR and scanned against the InterPro protein signatures database. We used Blast2GO to assign Gene Ontology (GO) IDs and EC numbers. GO IDs could be assigned to 45.61% of the input sequences, revealing that cellular and metabolic processes are the most represented among GOs (Figure S1B). Among enzymes, hydrolases were the most represented in the analyzed dataset, followed by transferases and oxidoreductases, and among them, peptidases formed the most important subclass (Figure S1C).

At last, Blast searches of viral sequences deposited at NCBI yielded 1031 CDS corresponding to 563 different viral proteins (Table S1). None of the viral hits corresponded to sequences previously described as involved in the production of viral particles in other braconid or ichneumonid species. Of these 1031 proteins, 1019 belonged to orthogroups comprising from two to 280 sequences from different parasitoid species. In addition, almost all displayed similarities with cellular proteins, which suggests that these CDS actually correspond to cellular genes that matched with virus acquired cellular genes. For the 12 sequences not assigned to an orthogroup, more than half had a collagen alpha domain and therefore could also be cellular proteins. All this indicates that none of these sequences was specific to *M. pulchricornis* and could be directly responsible for MpVLPs formation. 

### 2.3. Search for M. pulchricornis Genes a Priori Preferentially Transcribed in the Venom Gland 

Transcript levels of each protein-encoding sequence identified in the venom gland dataset were represented by the read coverage estimated by transcripts per kilobase million (TPM) to account for variability in CDS sequence length. For all 16,828 protein-encoding CDSs, TPMv (v for venom) ranged from 0.04 to 70,872 (Table S1), corresponding to a total number of reads ranging from 3 to 8,567,976. Only a minority of sequences (>1%) had a TPMv > 1000 indicative of a high level of transcription (Table 1; Table S1). The vast majority (∼96%) had a TPMv < 100, suggesting they were relatively poorly expressed in the *M. pulchricornis* venom gland (Table 1). 

To assess which sequences may be preferentially transcribed in the venom gland of *M. pulchricornis* compared to other tissues, the sequences were first used to search a previously published RNAseq obtained from antennae tissues [26]. Among the 16,828 CDS sequences in the venom, 16,091 (95.6%) were also found in the antennae. However, the corresponding TPMs in antennae (TPMa) were highly variable, ranging from 0.029 to 18,499 (Table S1). 

Of the 39 CDSs that were highly expressed in the venom gland (TPMv > 5000), 37 had a low coverage in the antennae (TPMa < 200), suggesting that they potentially correspond to genes more specifically transcribed in the venom gland compared to antennae. Contrariwise, the CDS most expressed in the antennae were poorly expressed in the venom gland and encoded odorant binding proteins or odorant-binding related proteins, which are proteins that play an important role in olfaction and are known to be abundant in olfactory organs [27]. Only four of these 39 CDSs had a match in the NCBI NR database with a protein of known function (Table 2; Table S1) and 12 had similarities with genes previously identified in the EST library of the venom gland of *M. pulchricornis* [24]. Most of the highly expressed CDSs (*n* = 24) belonged to orthogroups composed mostly by sequences from *M. pulchricornis* although they also contained sequences from other parasitoid species (Table 2). 

The most highly expressed CDS in the venom gland were contigs 60.p3, 64, 32 and 338. All four encoded proteins with similarities to hypothetical proteins previously identified in the *M. pulchricornis* venom gland [24]. Homologs were only identified in the transcriptome of *L. javana* venom gland for contig 60.p3, 64 and 338 (Table S1). Contig 60.p3 and contig 338 encoded a 55 AA-long and a 136 AA-long sequences, respectively, that matched with the hypothetical 136 AA protein from *M. pulchricornis*. The product of contig 338 was 79% identical to BAL70305.1 (E = 6 × 10^−70^) while that of contig 60.p3 corresponded only with the N-terminus (including a putative signal peptide) of BAL70305.1. Contig 64 encoded a 123 AA sequence protein 96% identical to the C-terminus of the hypothetical protein BAL70301.1 of *M. pulchricornis*. Finally, contig 32 encoded a protein of 172 AA that was 97% identical to BAL70307.1 (10^−115^) of *M. pulchricornis*. 

Among the CDSs with high TPMv values, four can be pointed out because of their high TPMv/TPMa ratio, suggesting that they are highly overexpressed in the venom gland relative to the antennae: contigs 52, 21, 155 and 49. Contigs 52 and 49 both matched with 97% identity with the hypothetical BAL70302.1 protein from *M. pulchricornis*, contig 21 to a 5′-nucleotidase (5NUC-like protein) from *A. cerana,* whereas contig 155 had no hit in the NCBI NR database.

### 2.4. Proteomic Analysis

**Figure 3:**
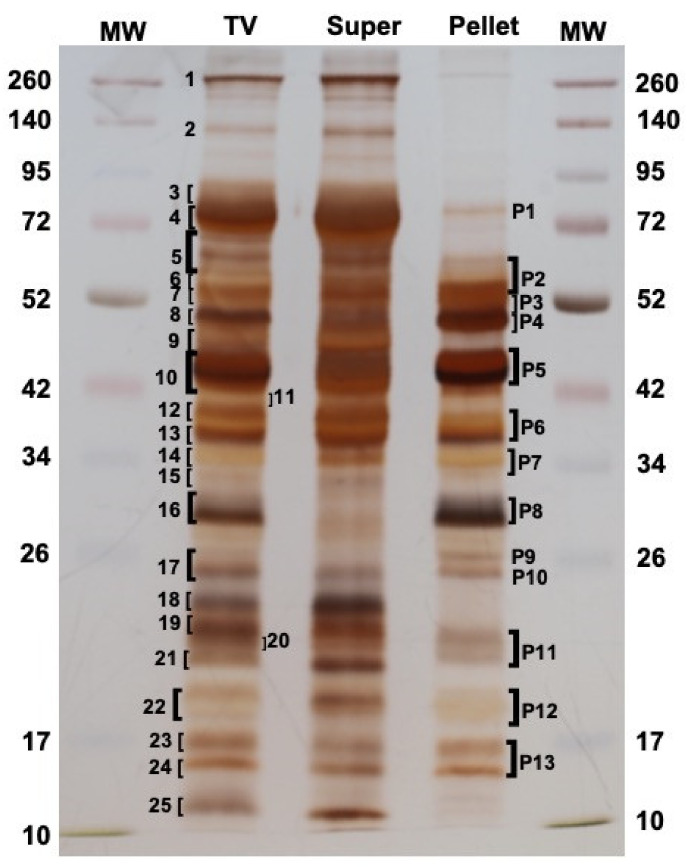
Protein profile of total venom (TV) compared to the supernatant (Super) and the MpVLPs pellet fractions obtained after centrifugation. A total of 25 and 13 bands were cut off for TV and the washed 15,000 g Pellets (MpVLPs) lanes, respectively, to be submitted to the MS-MS analysis. Left and right lanes correspond to molecular weight markers (MW in kDa). 12% SDS-PAGE in reducing conditions and gel was silver stained.

To define exactly which proteins were present in *M. pulchricornis* MpVLPs, the total venom (TV) was extracted from venom reservoirs and analyzed directly, or fractionated by centrifugation at 15,000× *g* into supernatant (Super) and pellet, with the later fraction containing only purified MpVLPs (see Figure 1F). When the proteins of each sample were separated by SDS-PAGE (Figure 3), some of the bands present in TV were strongly decreased or absent in the supernatant fraction while they were highly enriched in the pellet fraction of MpVLPs (such as bands P4, P5, P8, P9, P10, P11 and P12), indicating that MpVLPs had a specific protein profile.

Following separation by SDS-PAGE, a number of major protein bands were selected from the TV and MpVLPs samples (bands 1 to 25 for TV lane, bands P1 to P13 for Pellet lane; Figure 3). These bands were cut off from the gel, digested with trypsin, then submitted to mass spectrometry (see Materials and Methods). Protein identification was carried out using MASCOT searches against predicted translated contig sequences from the venom gland transcriptome. For TV, the peptides matched with 1354 different potential proteins and for MpVLPs with 516 different proteins. For subsequent analyses, we chose to focus on the 60 proteins with the highest Mascot proteomic scores (correlated to protein abundance according to spectral counting method, see material and method) for each of the two samples. Of these 120 sequences, 27 were common to both TV and MpVLPs samples, resulting in a total of 93 unique proteins. Note that because the TV sample included both MpVLPs and soluble proteins, we considered as “MpVLPs proteins” those that were enriched in the MpVLP sample plus those that were common to both samples (*n* = 60). Proteins present only in the TV were considered “soluble venom proteins” (*n* = 33).

BlastP similarity searches against the NCBI NR database indicated that most of the identified proteins had no matches or corresponded to proteins with no predicted functions (see Table 3 and Table 4 below). However, a large proportion of MpVLPs proteins has a predicted metalloprotease domain, while a large number of soluble venom proteins has a domain of Unknown Function 4803 (DUF4803). No hit was returned when MpVLPs peptides were blasted to the virus protein databank at Uniprot (Viralzone including polyDNAviridae).

### 2.5. The MpVLPs Proteins

The most represented protein family in MpVLPs was formed by the 32 sequences with correspondence to metalloprotease-like proteins (Table 3). This group included proteins with predicted metalloprotease domains, with similarities to the BAL70303.1 protein from *M. pulchricornis* (which includes a cysteine-rich ADAM domain), or sequences with no clear domain homology, but which were clustered in an orthogroup containing metalloproteases (Table 3).

However, based on Mascot scores, the most abundant proteins in MpVLPs were those encoded by contig 38 and contained a DUF4803 (Table 3). Contig 38 contained two proteins (named 38.p1 and 38.p2) in tandem on the same reading frame separated by an insertion. Both were DUF4803 containing proteins, and for both a high peptide coverage was obtained by MS-MS (Figure S2) explaining the very high Mascot score for this contig. One of the CDSs (38.p1) had a very high TMPv while the second one (38.p2) was tenfold less expressed. These two proteins belonged to two different orthogroups but showed 31% identity (E = 2 × 10^−60^) and both had a predicted C-ter transmembrane domain. They were also related to proteins of unknown function from different species of parasitoid wasps, although with low E-values (from E = 2 × 10^−12^), which may be due to the fact that all contained the DUF4803 domain. Two other proteins have DUF4803 domains (contig 72 and 897), making a total of four proteins of this family detected in MpVLPs. The third most abundant protein is encoded by contig 35. This unknown protein of 349 amino acids had no predicted signal peptide but a putative C-terminus transmembrane domain. The fourth and fifth most abundant MpVLPs proteins were encoded by contig 29 and 39, respectively. These proteins had no known function, domain, or peptide signal. The protein in contig 29 encoded a full-length protein that was 93% identical to BAL70308.1, and the protein in contig 39 was 98% identical to BAL70290.1 but has also some identity with BAL70306.1 (34% identity; E = 2 × 10^−19^) and BAL70304.1 (34%; E = 4 × 10^−10^). The abundant MpVLPs proteins also included proteins without a match in the NCBI database of proteins but having a predicted domain (3 with Calycin/Lipocalin domains and one with Serine protease inhibitor SERPIN domain).

Only four of the MpVLPs proteins have a clear match in the NCBI NR database. This is the case for a pancreatic lipase-like (contig 554), or a hyaluronidase (contig 293). Contig 6.p3 (and also 6.p2) and contig 1039 correspond to fibrillin-like proteins; they were about 35% identical to each other and all three have a predicted signal peptide. Fibrillins are high molecular weight secreted cysteine-rich glycoproteins (about 350 kDa) that contain numerous EGF-like calcium-binding domains [28]. The *M. pulchricornis* proteins corresponded to fibrillins mainly because of the high number of cysteine residues in their sequences (24 conserved cysteine positions out of the 29–31 cysteines of these sequences). Whether these proteins are derived from a fibrillin or are individual cysteine-rich proteins remains to be determined.

At last, all MpVLPs CDSs for proteins found in proteomics showed medium to high TPMv and were also expressed in antennae, although at a much lower level (TPMa ranging from 0.3 to 131). For nine of the twelve first MpVLPs proteins, a good correlation could be observed between the level of expression in the venom gland (TPMv > 5000) and the Mascot scores. Interestingly, the most abundant proteins in the purified MpVLPs were also the most abundant in the TV sample, indicating the importance of the vesicles and their content in the venom composition.

### 2.6. The Venom Soluble Proteins

Soluble proteins are those that are highly enriched in the TV sample compared with MpVLPs and represented 33 proteins (Table 4). Of these, 10 contained a DUF4803 domain and one, although lacking the domain, had a high identity to the venom protein 2 of *Microctonus hyperodae* Loan, which is also a DUF4803-containing protein. Four proteins were tagged metalloproteases or belonged to an orthogroup of metalloproteases, including a member of Neprylisin (contig 6971). Two proteins had a lipocalin domain and one could be an odorant binding protein (OBP) that are from the same protein family. Two matched with a pancreatic lipase-related protein, two with apolipophorins. One corresponded to a 5′ Nucleotidase (5NUC), one to a γ-glutamyltransferase (GGT), one to a cysteine-rich secretory proteins, antigen 5, and pathogenesis-related 1 proteins (CAP) domain protein. Several of the CDSs matched with proteins generally considered to be cytoplasmic proteins such as glyceraldehyde-3-phosphate dehydrogenase, 14-3-3 protein, myosin, aldose reductase and arginine kinase.

A surprising result was that, in contrast to MpVLPs, many of the contigs encoding the most enriched soluble proteins had low TPMv, and in some cases even appeared to be more expressed in the antennae than in the venom gland (i.e., arginine kinase and OBP). Based on their TPMv values, three groups of venom-soluble proteins could be defined: those with TPMv > 1000 (10 contigs) and those with TPMv < 100 (16 contigs), with the remaining seven contigs ranging between 112 and 581. For example, almost all CDSs encoding DUF4803 domain-containing proteins were weakly expressed in the venom gland (except contig 275). Conversely, those encoding metalloproteases, even with a low Mascot score, were highly expressed with the exception of neprilysin whose expression was very low in both venom gland and antennae.

The most expressed CDS was contig 21 encoding the 5NUC-like protein, while among the least expressed were those encoding GGT, OBP, myosin, arginine kinase, neprylisin and apolipophorins. Some of these proteins such as arginine kinase, OBP and apolipophorins play an important role in lipid transport and immune response in insects and usually circulate in the hemolymph [29,30]. Their presence in the venom could therefore result from a contamination during venom collection, yet other classically abundant hemolymph proteins (i.e., HSP chaperones and phenoloxidases) were absent from the data. Another possibility is that these proteins entered passively or were concentrated in the venom by a specific transport process, but this remains to be ascertained. Another possibility for some of these proteins is that, because the venom is enclosed and separated from the rest of the body fluids, the released cellular proteins may have accumulated in this fluid. However, this does not exclude that these proteins may play a role in the envenomation. For example, arginine kinase, which plays a critical role in maintaining insect cellular energy homeostasis, has been found in almost all hymenopteran venoms. Once injected by the wasp, it can modulate the host extracellular ATP/ADP ratio, which may significantly affect the host purinergic signaling-activated innate immune response [31].

### 2.7. The Most Represented Venom Proteins: Metalloproteases and DUF4803 Proteins

#### 2.7.1. The Venom Metalloproteases

Metalloproteases were found in large numbers in the transcriptomic analysis of the *M. pulchricornis* venom gland and a total of 36 putative metalloproteases were found by proteomics. The majority (*n* = 32) were enriched in MpVLPs. In addition, a neprylisin-like protein (contig 6971) was detected in the soluble fraction and will be discussed in more details below.

Metalloproteinases are toxins described in the venom of almost all venomous animals [32] and also used as virulence factors by bacteria [33]. The best studied example for the role of these enzymes in envenomation is that of snake bites where injected metalloproteinases interfere with the hemostatic system, resulting in hemorrhage [34]. Snake venom metalloproteinases also induce local myonecrosis, skin lesions, and an inflammatory response [35]. Metalloproteases have also been identified in the venom of hymenopteran wasp and bee species [36,37,38,39]. These metallopeptidases have been suggested to have a wide range of functions, including nutrition, suppression of host cellular defense, and degradation of host defense molecules.

Metalloproteases include a number of families that differ in their structure and catalytic domains. A disintegrin and metalloproteinase (ADAM) and a disintegrin and metalloproteinases with thrombospondin motif (ADAM-TS) are normally multidomain proteins of more than 800 AA that typically comprise: (i) a signal peptide, (ii) a pro-domain, (iii) a catalytic domain with a reprolysin-type zinc-binding motif (HEXXHXXG/N/SXXHD), and (iv) a disintegrin-like domain. The disintegrin domain may be followed in some proteins by an ADAM-cysteine rich (ADAM-CR) domain and a C-terminus of varying length [40,41,42]. The different domains can be cleaved from the catalytic domain after secretion to give rise to the mature protein [43]. Collagenase/matrix metalloproteinases (MMPs) are also multidomain proteins and can have a lower molecular weight ranging from 25 to >100 kDa. The metal ligands and active site of MMPs show a similar HEXXHXXGXX motif [44] and their substrate and cleavage sequence specificity overlap with that of ADAMs. However, some metalloproteases, for instance snake venom metalloproteinases (SVMPs), can be much shorter. Indeed, SVMPs can be divided into three classes: Class I SVMPs range from 20 to 30 kDa and contain only a pro-domain and the proteinase domain. Class II is 30–60 kDa, contains the pro, proteinase and disintegrin domains and Class III, 60–100 kDa, contains the pro, proteinase, disintegrin-like and cysteine-rich domains structure [45,46].

The putative metalloproteases sequences from *M. pulchricornis* venom ranged from 126 AA (contig 379.p2) to 592 AA (contig 375) suggesting that some sequences may be incomplete or represent the N- and C-terminus of the same protein. They shared from zero to about 70% identity, certainly due to differences in sequence length but nonetheless suggesting a high divergence among them (Figure S3). A putative N-terminus Methionine could be assigned to 18 sequences and a predicted signal peptide sequence to 11 of them (Table S3). Despite their variable length, most of the *M. pulchricornis* predicted metalloproteases have a labeled catalytic domain such as collagenase (matrix metalloprotease; MMPs), A disintegrin and metalloproteinase (ADAM, ADAM-TS, reprolysin) or an ADAM-cysteine rich domain (ADAM-CR) (see Table S3). Of the 36 metalloprotease sequences, six had the canonical zinc-binding/catalytic site motif and 17 had a more or less degenerate motif. Most sequences had a furin cleavage site in their N-terminus between the pro- and the catalytic domain and the conserved triad of amino acids (E-D-N) involved in the first Ca^2+^ binding site of ADAMs (Figure S3). In addition, 22 of them showed a putative ‘Met-turn’, downstream of the catalytic site position, although not always in the V/IMA/S canonical motif but mainly L/IMD/Q motif [42]. After this Met-turn, 24 of the sequences showed the conserved proline marking the end of the metalloproteinase domain and then their C-terminus region contained up to seven conserved cysteine residues suggesting the presence of a disintegrin/cysteine-rich domain. None of these sequences have other EGF, transmembrane or cytoplasmic domains and are therefore structurally closer to class II SVMPs. Interestingly the 121 AA sequence from *M. pulchricornis* BAL70303.1 that contained 10 Cysteine residues aligned with this disintegrin/cysteine-rich domain in the other sequences, suggesting it may be an incomplete sequence that contains only this domain like our contigs 379 and 1198 (Figure S3). Whether an evolution leading to different classes of metalloproteinases as described in SVMPs has occurred in *M. pulchricornis* will necessitate to obtain the complete genome to have access to full length CDSs.

Several neprylisin-like CDSs from *M. pulchricornis* were distributed in different orthogroups, OG0017160 (7/7), OG0000177 (9/114), OG0000299 (12/99 contigs), OG0008057 (2/25), OG0002203 (1/49), OG0013455 (1/10), but only one neprilysin encoded by contig 6971 (named MpNEP) was found as a venom soluble protein. Neprilysins, also known as neutral endopeptidases, are normally membrane bound proteins, but their ectodomain can be released from the cell surface, producing a free circulating enzyme [47]. Such a mechanism may explain its presence in the soluble fraction of the venom. MpNEP had a low Mascot score and, as previously described, had a very low TMPv value in the venom gland (TPMv = 1) suggesting that it may be not synthesized in this tissue. However, since the protein had a conserved active site (Table S3) it could, even in small amounts, have an effect on the parasitoid’s host. NEP and NEP-like enzymes are involved in the processing of various neuropeptides and peptide hormones and play a role in the regulation of lipid and carbohydrate metabolism in insects [48,49,50]. Neprilysin-like enzymes have been commonly found in wasp parasitoid venoms [51,52,53] and a 94 kDa neprilysin−like protein (VcNEP) was found to be associated with virus−like particles produced in the calyx region of *V. canescens* [54]. This VcNEP has been suggested to induce cell adhesion and hemocyte spreading of the host *Ephestia kuehniella* Zeller. Contrariwise, injection of a recombinant neprilysin from the venom of the endoparasitoid wasp *Cotesia vestalis* Haliday (previously *Cotesia plutellae*) disrupted immune responses against *E. coli* in its host *Plutella xylostella* L. [53]. Although the role of neprilysin in parasitoid venoms remains to be clarified, this enzyme is known to play a role in the venom of snakes and solitary and social wasps, in the physiological clearance of natriuretic and vasodilatory neuropeptides [55,56]. Neprilysin-like enzymes present in spider venoms may also play a role in extracellular matrix degradation or cell apoptosis [57,58]. Similar effects of MpNEP can be expected in wasp hosts.

#### 2.7.2. The DUF4803 Containing Proteins

A total of 14 proteins identified in *M. pulchricornis* venom by mass spectrometry contained a predicted DUF4803 domain (Table S4). In addition, we identified one contig (1164) that encoded a CDS lacking the DUF4803 domain but that matched with DUF4803-containing proteins. On this basis, 11 DUF4803 proteins were enriched in the soluble fraction of venom while four were enriched in MpVLPs. These proteins belonged to 10 different orthogroups, four of which contained only two to 34 CDSs from *M. pulchricornis*, three were shared with only *L. javana,* and three with one or more other species (Table S1). The DUF4803 (PF16061; IPR032062) protein family contains only arthropods proteins. Among them, 83 proteins have been reported in Hymenoptera (wasps, bees, ants …) including 56 in parasitoids (33 in Chalcidoidea and 23 in Braconidae). However, DUF4803 proteins are also found in venom-less insects such as Drosophila, suggesting a ubiquitous role for this domain. Indeed, DUF4803 proteins are typically between 350 and 690 amino acids and can have several other domains such as immunoglobulin, protein phosphatase or zinc finger domains, suggesting potential different functions or localizations.

Comparison of the 15 MS-MS-validated *M. pulchricornis* DUF4803 venom protein sequences showed an identity ranging from 9% to 53%, suggesting a large divergence between them. Protein sequence alignment showed a specific motif of highly conserved cysteine residues (Figure S4), in particular the characteristic DUF4803 CxxCxCxC motif, yet however the other suggested canonical motif RRY was not well conserved. A VI/VTGIK/RF motif present at the end of the DUF domain is duplicated in the C- terminus of the sequences suggesting a partial duplication of the domain. None of these venom DUF4803 proteins have another known domain.

Although DUF4803 proteins were previously found in proteomics in the venom of parasitoid wasps of the braconid species such as *B. nigricans* [59], *Psyttalia lounsburyi* Silvestri [51] and *A. ervi* [60] the role(s) they may play in parasitic success remains to be understood.

### 2.8. Other Venom Proteins of Interest

#### 2.8.1. The 5′ Nucleotidase (5NUC)

**Figure 4:**
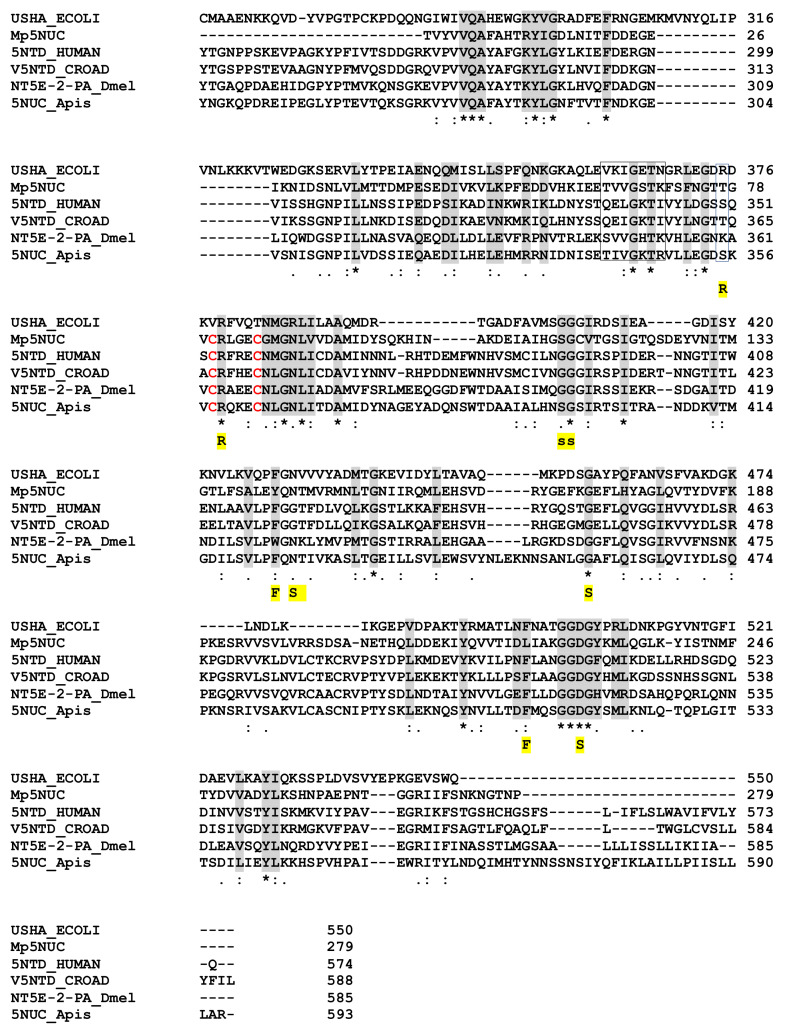
Sequence alignment of nucleotidases from *M. pulchricornis*, human, *E.coli*, snake, drosophila and bee. Important conserved residues or motifs are shaded in grey or boxed. Functionally important residues are marked below the alignment with the following labels: “R” indicates the three arginine in the active site of the *E. coli* enzyme; “F”, the phenylalanines that bind the adenine moiety; “S” the residues that interact with the substrate and “s” those involved in non-polar interactions (from [61]). The only two cysteines in MpNUC that can form a bond are in red. USHA.ECOLI, Protein UshA *Escherichia coli* P07024 (uniport); 5NTD.HUMAN, 5’-nucleotidase *Homo sapiens* P21589; V5NTD.CROAD, *Crotalus adamanteus* venom 5’-nucleotidase F8S0Z7; NT5E-2PA, *D. melanogaster* Q8SZY4; 5NUC.Apis, protein 5NUC *Apis mellifera* XP.394018 (NCBI). Identical amino acids are indicated by a star, conservation by a colon and substitution by a dot.

The 179 AA *M. pulchricornis* venom 5NUC protein encoded by contig 21 (which is 96% identical to the protein encoded by contig 19) found in the soluble fraction was found to be incomplete since it matched only the C-terminus domain of ecto-5NUC from bacteria, Hymenoptera and vertebrate sequences, including snakes and human CD73 (Figure 4). Therefore, the catalytic and ion binding sites were absent but some of the important features of the enzyme were retained [61]. The *M. pulchricornis* protein was not predicted to have a GPI anchor like human CD73 ecto-5NUC.

The 5′-nucleotidase is a ubiquitously distributed enzyme in eukaryotes and prokaryotes. It is commonly found in the venoms of bees, wasps and ants [39,62] and is present in the venom or used as a virulence factor by various other species. For example, in snake venom, this enzyme has an anticoagulant effect and inhibits platelet aggregation in humans. In animals, the cytosolic and extracellular 5NUC enzymes (which are bound to the membrane by a GPI anchor) are structurally unrelated [63,64]. There is also a soluble form of these ecto-enzymes that is shed from the membrane by the action of phosphatidylinositol-specific phospholipase. The ecto-5NUC catalytic domain of snake venom belongs to a superfamily of dinuclear metallophosphoesterases, which hydrolyze very different substrates, including phosphoproteins, nucleotides and nucleic acids. In humans, two 5NUCs, CD39 and CD73, have complementary activity: CD39 hydrolyses both ATP and ADP and produces AMP while CD73 uses AMP and generates adenosine [65].

Because in insects adenosine is involved in a broad range of physiological processes, including cell growth, differentiation and immunosuppression, 5’nucleotidase may have an immunoregulatory role when injected into the host by converting AMP to adenosine [61]. In *D. melanogaster*, extracellular adenosine is involved in the release of glucose from glycogen, a systemic metabolic switch required for effective resistance to pathogens [31]. 5NUC may have a different role in parasitoid wasp venoms but, based on the actions described, it may interfere with host immunity directly through adenosine production or depletion of circulating ATP or indirectly by modulating host metabolism to promote parasitoid larval development.

#### 2.8.2. The Calycin/Lipocalin Proteins

**Figure 5:**
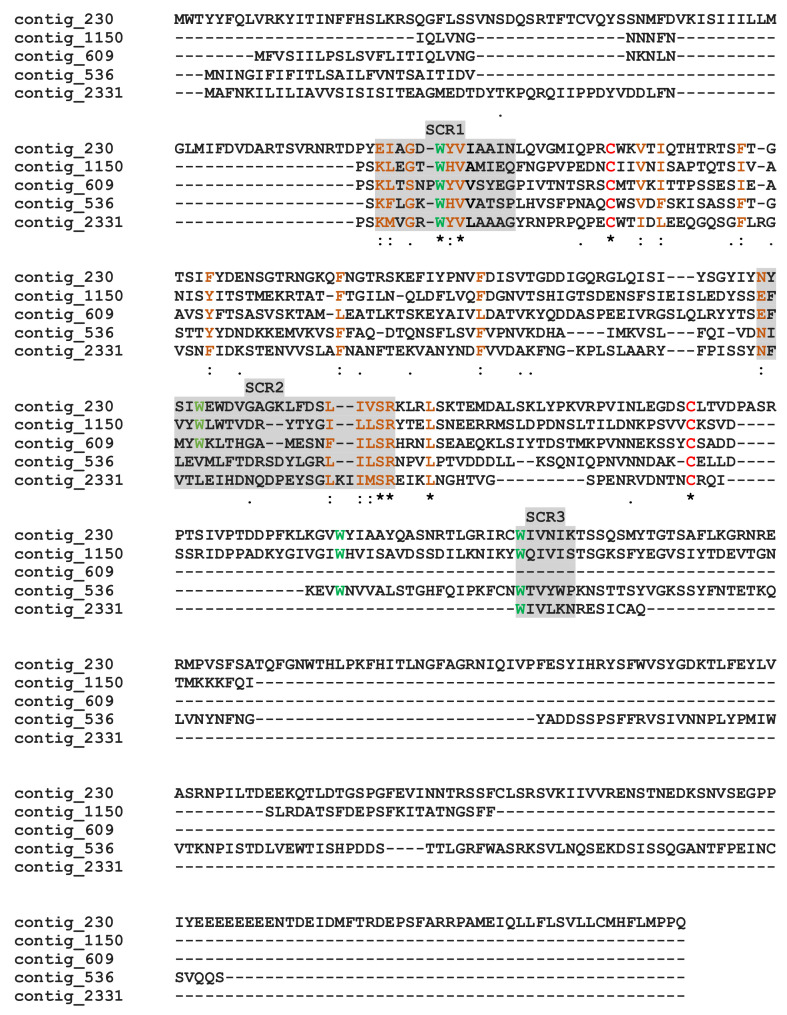
Alignment of the putative *M. pulchricornis* venom lipocalins. Grey boxes indicate the three putative lipocalin structural regions (SCR1, 2, 3). In green, the hydrophobic tryptophan (W) retrieved in SCRs, in red the cysteines. Identical amino acids are indicated by a star, conservation by a colon and substitution by a dot.

*M. pulchricornis* venom contained proteins with a calycins/lipocalin domain: three are enriched in the soluble protein fraction (contig 2331, 536 and 609) and two in MpVLPs (contig 1150 and 230). Calycins form a large protein superfamily consisting of a heterogeneous group of secreted proteins, including lipocalins and fatty acid-binding proteins (FABPs), which bind a wide variety of small hydrophobic ligands and exhibit high functional diversity [66,67]. Calycins/lipocalins are quite diverse and have little sequence identity, except for characteristic short conserved motifs (SCRs) that may be receptor-binding sites for hydrophobic compounds [68,69]. The *M. pulchricornis* sequences shared only between 15–33% identity, but when these sequences were compared to well-described lipocalins [69], the three putative SCRs could be located (Figure 5). In all lipocalins, SCR1 is the most conserved motif, and the *M. pulchricornis* sequences display the central SCR1 motif GXWH/Y found in classical lipocalins such as retinol binding protein [70]. The positions of the other two SCRs are indicated but are much less conserved (even in canonical lipocalin sequences). Some amino acid identities/similarities can be found in the SCR2 region and a degenerated SCR3 appears to be present only in the four longest sequences. The *M. pulchricornis* proteins also showed two classically conserved cysteines that may form a disulfide bond but lack the CXXXC motif found in OBPs (Figure 5).

Proteins of the lipocalin superfamily have been identified in the venom of the parasitoids *Chelonus inanitus* L., *Pteromalus puparum* L., and *N. vitripennis* and various other hymenopteran species. In *P. puparum,* OBP-like mRNA expression in the venom apparatus is upregulated after feeding and parasitism, suggesting a role in venom gland metabolism or host interaction (or both) [71]. The main ant venom allergen of *Dinoponera quadriceps* Kempf and the fire ant *Solenopsis invicta* Buren are OBP/PBP-like capable of triggering anaphylaxis [72]. The role(s) that lipocalins may play in the host after wasp venom injection remains to be analyzed, but this type of molecules, with their different functions such as retinoids, arachidonic acid and steroids transport, pheromone transport and prostaglandin synthesis, play many different roles in metabolism and physiological regulation [66,67]. Lipocalins are also members of the calycins superfamily, which includes avidins, a group of metalloproteinase inhibitors, and triabin, which is found in several groups of hematophagous arthropods and has various anti-hemostatic functions by interfering with the assembly of procoagulant complexes, preventing platelet activation and aggregation, and sequestering amines such as serotonin (for review [32]).

To this group of lipocalins proteins we can add the protein encoded by contig 267 with a juvenile hormone binding protein domain (JHBP) found in MpVLPs. This protein did not blast with known JHBPs but with several parasitoid proteins with tandemly repeated JHBP domains. The sequence also lacked the cysteine residues forming the two bonds in the JHBP proteins. The CDS of contig 267 has a TMPv of 2950 suggesting high expression in the venom gland. In the hemolymph, the JHBP protein transports Juvenile Hormone from the sites of its synthesis to target tissues and protect it from hydrolysis. Juvenile hormone (JH) has a profound effect on insect embryogenesis, larval development and reproductive maturation of adult forms. Once injected, this protein can bind to the host JH to block its development and molting. However, since *M. pulchricornis* contig 267 does not have the classical characteristics of the JHBP protein and the structure of the JHBP domain resembles that found in some mammalian lipid-binding tandem-repeat proteins that increase bacterial permeability, it cannot be ruled out that this protein may have one of these other roles.

#### 2.8.3. The GGTs

A γ-glutamyltranspeptidase (GGT) was found with a high score in soluble venom proteins. Among the proteins present in braconid venom, it is suggested that a GGT play an important role in the success of the parasitoid *A. ervi*: once injected, venom GGT1 can target the reproductive tract of the female aphid host, leading to ovarian degeneration and decreased fertility [73]. GGTs are normally cell surface hydrolases that cleave glutathione and other γ-glutamyl compounds and are essential in cysteine homeostasis, but like neprilysin, they can be cleaved and released into the surrounding environment [74].

**Figure 6:**
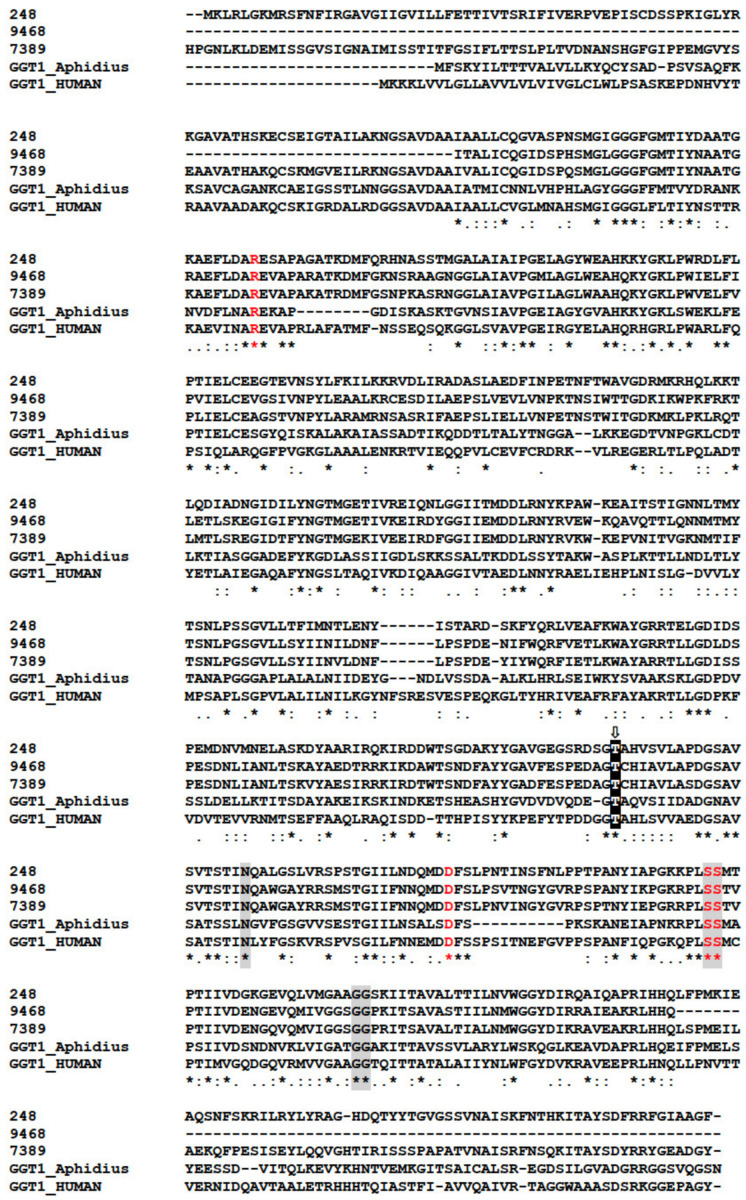
Clustal alignment of the *M. pulchricornis* GGTs with the human (CAG30380.1) and *A. ervi* (CAL69624.1) GGT1 sequences. The catalytic threonine residue that forms the N-terminus of the small subunit (T381) after autocleavage to form the mature heterodimeric enzyme is highlighted with a black box containing a white “T” with an arrow above. Residues proposed to interact with glutathione in human GGT1 are shaded in grey and residues that significantly reduced human GGT enzymatic activity by site-directed mutagenesis are in red. Identical amino acids are indicated by a star, conservation by a colon and substitution by a dot.

In our analysis, GGTs formed the orthogroup OG0000441 (Table S1) encompassing 87 sequences from various Hymenoptera including six from *M. pulchricornis*. Five of the six *M. pulchricornis* sequences blasted with high e-values with GGTs from the braconid *M. demolitor* (NCBI ID: XP.008555551.1) and four of these GGTs sequences were found in the *M. pulchricornis* venom proteome (CDSs from contig 11339, 248, 9468, 7389). Two of the encoded proteins were 100% identical (contig 11339 and 248). The other two were 80% identical to each other and 62% (for 9468) and 55% (for 7389), respectively, with the 11339/248 CDS sequence. The three sequences were compared with those of GGT1 from *Aphidius ervi* venom and human GGT1 (Figure 6). The main feature of the GGTs was retrieved in the *M. pulchricornis* sequences, suggesting that they function like GGT1 in venom and may have a similar function to that in *A. ervi* venom. However, none of the GGTs in the *M. pulchricornis* sequences had AA mutations that have been found in some *A. ervi* venom isoform sequences, mutations that reduced the enzymatic activity of human GGT [60]. Interestingly, a very high number of OG0000441 members (49/87) were predicted in the transcriptome of the venom gland of the ichneumonid *Pimpla turionellae* L., suggesting strong GGT amplification in this species.

#### 2.8.4. Pancreatic Lipase Like Proteins

**Figure 7:**
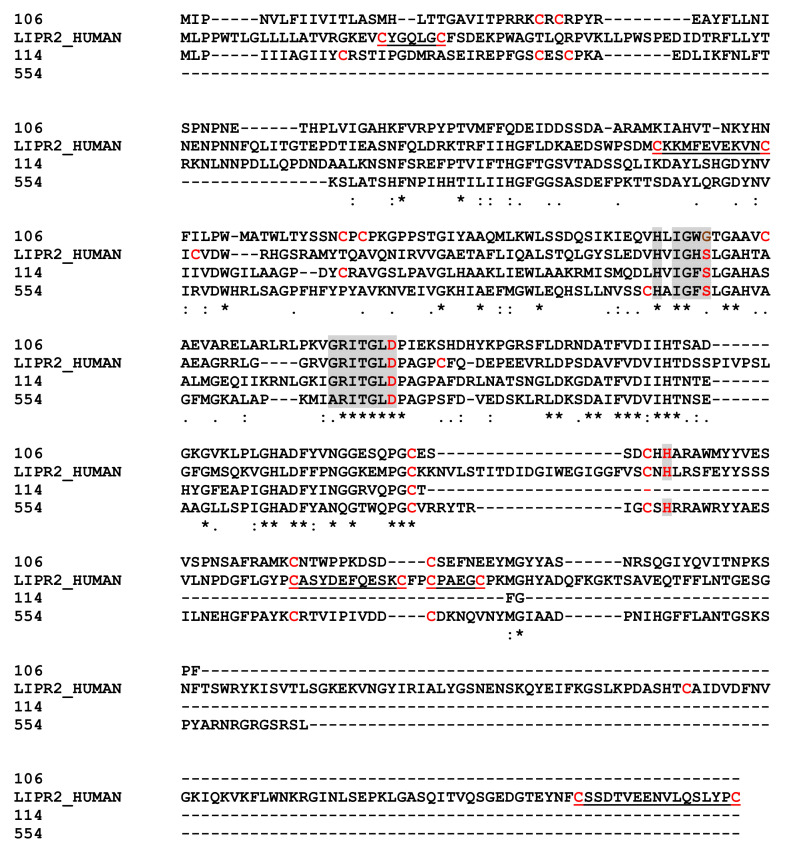
Sequence Alignments for Pancreatic Lipase Sequences. Key active site residues from Human Pancreatic lipase-related protein 2 (P54317) are in red shaded in grey; in shaded grey, amino acids conserved around the active site residues. The underline human sequence residues represent the two bonds between cysteines. Identical amino acids are indicated by a star, conservation by a colon and substitution by a dot.

One soluble protein and one in MpVLPs matched with pancreatic lipase-relative protein 2-like (PLRP2-like) and one soluble protein with a triacylglycerol lipase. These sequences had 35% (contig 106 versus 114 and 554) to 49% (114 versus 154) identity. When compared with human pancreatic lipase-related protein 2/PNLIPRP2 (Figure 7) the three important active site residues (S,D,H) could be retrieved for at least two of the three sequences, with the first serine replaced by a glycine in the contig 106 protein [75]. While at least four of the cysteine residues were conserved, most of those involved in disulfide bonds in the human protein were absent. Lipases are widely distributed in animals, plants and prokaryotes. Pancreatic lipases (EC 3.1.1.3) are one of the three tissue-specific isozymes described in higher vertebrates [75]. They hydrolyze long chain triacyl-glycerol to free fatty acids and monoacylglycerols at the lipid–water interface and therefore play a crucial role in controlling lipid uptake, transport and utilization. Members of the lipase family have been found in the venom of insects including social Hymenoptera (bees, wasps and ants) and various other species such as snakes. In general, these major lipases were from the phospholipase-A family, which uses a different catalytic mechanism to hydrolyze acyl-ester bonds of phosphatidylcholine.

In vertebrates, PLRP2 enzymes are mainly digestive. These enzymes participate in the hydrolysis of triglycerides, phospholipids and vitamin A esters and also have a high activity on monogalactosyl diglyceride [76], the major lipids of plant cells. Recently PLRP2-like proteins have been described in the ectoparasitoid wasp *P. puparum*, and for four of them peptides were found in the venom proteome [77]. Based on the functions of PLRPs, PLRP2-like found in *M. pulchricornis* venom may contribute to hijacking host lipid metabolism for the benefit of endoparasitoid eggs/larvae.

#### 2.8.5. Hyaluronidase

**Figure 8:**
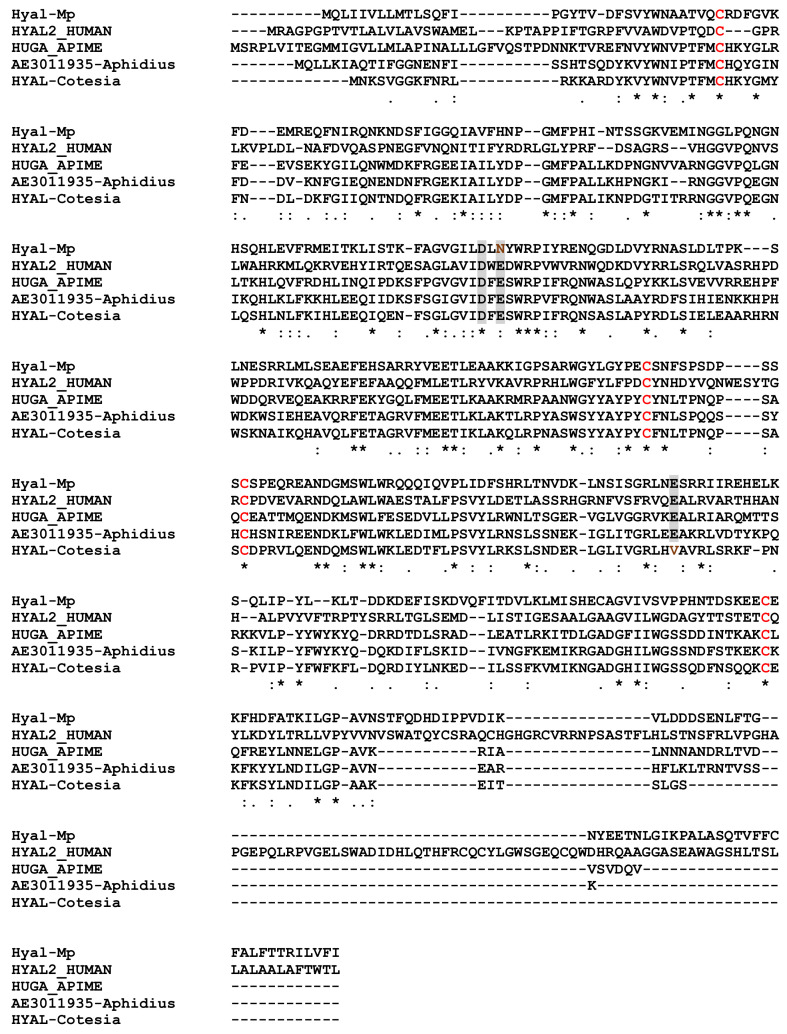
Alignment of hyaluronidases from *M. pulchricornis* (Hyal-Mp from contig 293), Human (Hyaluronidase 2, HYAL2-HUMAN, Q12891), bee (*Apis mellifera*, Q08169) and *Cotesia congregata* (CCQ71107). The putative active-site residues Asp(D), Glu(E), and Glu(E) (shaded in grey) of the hyaluronidase were conserved in the *M. pulchricornis* sequence, although a conservative replacement E → N occurred that may affect its activity [81]. The four cysteine residues forming two disulfide bridges are also conserved (in red). Identical amino acids are indicated by a star, conservation by a colon and substitution by a dot.

A hyaluronidase protein was present in MpVLPs although its activity may be reduced due to an AA replacement in the active site (Figure 8). Hyaluronidases are found from bacteria to mammals. In mammals, these enzymes catalyze the hydrolysis of hyaluronan (HA) and chondroitin sulphates from the extracellular matrix. This property of increasing tissue permeability has led to the suggestion that hyaluronidases present in the venom of bees, stinging wasps, snakes and other species, along with phospholipases, facilitate the diffusion of other venom components throughout the body [78,79]. Although HA is widely distributed in vertebrates, it has apparently not been found in invertebrates, including insect species [80]. Therefore, the potential role of hyaluronidase in host after parasitoid oviposition requires further attention, including whether it can act on other glycosaminoglycans components of the insect matrix such as chitin, chondroitin or chondroitin sulfate, which can be degraded by hyaluronidase [80].

#### 2.8.6. CAP Domains Protein

**Figure 9:**
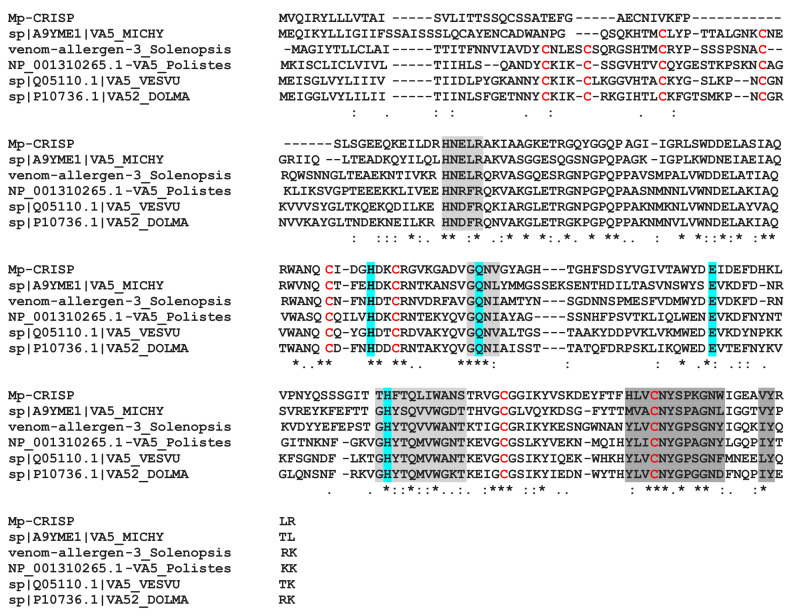
Alignment of the first CAP domain of contig 2455 (Mp-CRISP) with selected sequences of hymenoptera antigen 5 allergens. The CAP signature motifs are shaded in grey and conserved residues that form the putative active site are shaded in cyan [85]. Cysteine residues that form disulphide bridges in Ag5 proteins are marked in red. Asterisks, colons and periods indicate identical, conserved and semi-conserved residues, respectively. Sequence from *Vespula vulgaris* (VA5.VESVU, Q05110.1), *Dolichovespula maculata* (bald-faced hornet)(VA52.DOLMA, P10736.1), *Polistes dominula* (European paper wasp) (VA5.polistes, NP.001310265.1), and *Microctonus hyperodae* (A9YME1.1).

The MpVLPs protein from contig 2455 contains three CAP domains (cysteine-rich secretory proteins or CRISPs), antigen 5 (Ag5), and pathogenesis-related 1 (PR-1) proteins). CAP domain proteins are distributed in a wide range of organisms and have a wide range of functions [82]. CRISPs are found in the majority of snake venoms where they disrupt prey homeostasis through several mechanisms, including inhibition of ion channels and angiogenesis [83]. In snakes, CRISP genes have undergone accelerated evolution aided by strong positive selection and directional mutagenesis [84]. PR-1 proteins are ubiquitous in plant species and it is suggested that PR-1 has a broad antimicrobial function. Antigens 5 are proteins of unknown function in Hymenoptera venoms with strong allergenic potency [85]. When we aligned the first CAP domain of contig 2455 with the Hymenoptera antigen 5 sequences (which have only one domain), we obtained a good match with about 35–40% identity (Figure 9). To date, the role of CAP domain proteins in parasitoids venom is unknown.

#### 2.8.7. The SERPIN

**Figure 10:**
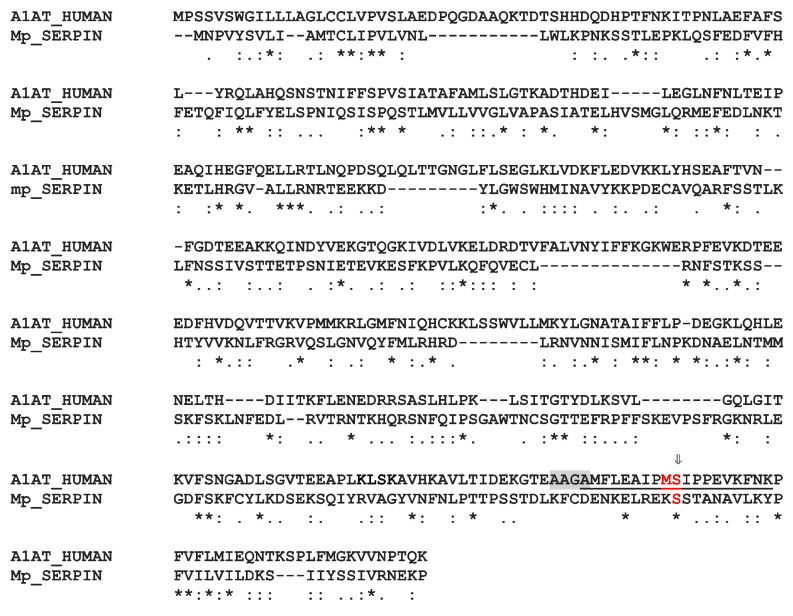
Amino acid sequence alignments of contig 590 protein (Mp_SERPIN) and human SERPINA1 (P01009|A1AT_HUMAN Alpha-1-antitrypsin). The RCL position of the human sequence is underlined. The cleavage site is situated between the position P1 (Met) and P1′ (Ser) of the human sequence, only the Ser is conserved in Mp_SERPIN. The important Alanine-rich hinge site shaded in grey is also absent from contig 590. Asterisks, colons and periods indicate identical, conserved and semi-conserved residues, respectively.

The MpVLPs contig 590 encodes a serine proteinase inhibitor (SERPIN) domain protein. SERPINs participate in a suicide inhibitory mechanism that permanently inactivate proteinases [86]. They share a conserved core structure critical for their inhibitory function. The structure of contig 590 corresponds to α-1-antitrypsin, the human prototype being SERPINA1. In the native conformation, the reactive center loop (RCL) of SERPINs, a short flexible strand recognized by proteases, is exposed for interaction. After recognition, an initial noncovalent complex is formed and then a covalent bond is established upon cleavage of the peptide bond between AA P1 and P1′ (see Figure 10). Besides Ser P1′, only one other AA is conserved in the *M. pulchricornis* RCL compared to human SERPINA1. The conserved alanine rich hinge important for the inhibitory function of SERPINs [86] is also absent suggesting that SERPIN from contig 590 is potentially non inhibitory. The role of SERPINS in arthropods has been studied mainly for their functions in innate immune responses [87]. For example, SERPINs are involved in the phenoloxidase activation cascade and inhibition of this cascade by a *Leptopilina boulardi* Barbotin, Carton & Kelner venom SERPIN, LbSPN, is part of the success of this wasp in some Drosophila species [88]. Interestingly, in two well-studied strains of *L. boulardi*, LbSPN retain its active RCL in one strain but not in the other [20], suggesting, as with *M. pulchricornis,* a possible different role for this protein than protease inhibition.

## 3. Conclusions

As in many parasitoid wasp venoms studied to date, the most represented class of proteins in *M. pulchricornis* venom corresponded to “uncharacterized” proteins or proteins with no hit in the database. Although a number of these *M. pulchricornis* proteins have already been described in the venom glands of other venomous species, they currently have no determined function. Most of the known proteins or proteins with a known functional domain have also been found in the venom of different species and may have the same suggested role, keeping in mind that in most cases the described role is a defensive one against a vertebrate organism with a different physiology from that of the host insect caterpillar.

Overall, the main conclusions of the present study are (i) the relative preponderance of metalloproteases in the MpVLPs and of DUF4803 domain proteins in the soluble fraction of the venom, and the high diversity of these two main families of venom proteins produced in venom glands, (ii) the lack of evidence of a viral origin for MpVLPs.

Zinc metalloproteases are abundant in *M. pulchricornis* and particularly well represented in MpVLPs. Metalloproteases are common in parasitoids venoms, and a number of studies have been done to understand their role. One example is the venom metalloprotease from *Microplitis mediator* (named VRF1): the C-terminus fragment, which contains the catalytic domain, enters the hemocytes of the host *Helicoverpa armigera* where it cleaves the NF-κB Dorsal factor, a process related to the modulation of wasp egg encapsulation by the host [89]. In the ectoparasitoid wasp *Eulophus pennicornis*, three genes encoding metalloproteinases with a C−terminus reprolysin domain are expressed in the venom gland and injection of one of these recombinant metalloproteases into the larval host *Lacanobia oleracea* resulted in partial mortality of the insects, with the surviving ones exhibiting delayed development and growth [37]. In *N. vitripennis*, an in vitro study indicated also that venom metalloproteases may be involved in apoptosis of insect cultured cells [90]. From these examples, and based on the elevated number of metalloproteases incorporated into MpVLPs, it could be suggested that MpVLPs are a sort of “metalloprotease bomb” that, once injected into the host, enter host immune cells or other cells to impair the immune response and/or development as well as other physiological functions. This is consistent with the reported in vitro effect of MpVLPs in inhibiting the attachment and the spreading of host hemocytes and ultimately inducing their apoptosis [14,15].

Another family of proteins that is abundant in the venom of *M. pulchricornis*, particularly in the soluble fraction, is the DUF4803 containing proteins family. Unfortunately, no functional information is currently available for these proteins, which are also present in the venom of other braconid species. Therefore, these proteins most probably deserve to be studied in more detail in the future in order to determine their function in parasitoid venoms.

Our study showed that members of the metalloprotease and DUF4803 families are highly represented in the venom proteome of *M. pulchricornis* and in the transcriptome of the venom gland. In Hymenoptera and other venomous taxa, the venom protein cocktail is classically enriched through gene duplication, thereby increasing its functional divergence through evolution of new functions or neofunctionalization [32,91]. One of the best examples is the snake venom family of metalloproteinases: in some species, these proteins have expanded massively from a single copy to 31 genes in tandem through gene duplication events [46]. This expansion has been accompanied by mutations within the active sites, suggesting that some proteins have lost their metalloprotease function and may have evolved other functions. The evolution of venom genes through processes including multi-functionalization, co-option, and gene duplication has also been suggested in parasitoids [92]. Therefore, similar types of evolutionary mechanisms may have occurred in the lineage of *M. pulchricornis* resulting in an increase of the diversity of its venom proteins, and/or potentially the appearance of new functions for some of the venom proteins. Studies of species related to *Meteorus* species will be of interest to resolve this issue.

Finally, our study did not find any candidate viral gene or protein that could be linked to the production of MpVLPs. The approach undertaken here is similar to that performed in species producing virus-derived particles such as PDVs or VLPs in their ovarian calyx. Indeed, in these models, the endogenous virus was detected both by the transcriptomic analyses of the producing tissue and by the proteomic analyses of the purified particles. In the present work, we did not find any specific viral gene expressed in the venom gland nor a viral protein in MpVLPs. The hypothesis is therefore that either *M. pulchricornis* VLPs are formed by a viral machinery derived from an as yet undetermined virus and therefore whose sequences are absent from public databases, or that MpVLPs are produced directly by a specific pathway in the venom gland cell that remains to be deciphered.

## 4. Materials and Methods

### 4.1. Insects

*M. pulchricornis* cocoons (about 10 cocoons) were recovered from *Spodoptera* sp. larvae collected in 2015 in the South of France. Mated adult females were used to parasitize larvae of a laboratory strain of *Spodoptera littoralis* Boisduval. Parasitized hosts were reared under a long-day photoperiod (16 h light: 8 h dark) at 25 °C until emergence of wasps. Female wasps used in the different experiments were at least 3 days-old. The reproductive apparatus was obtained from cooled anaesthetized female wasps by traction on the ovipositor and venom glands separated by dissection under a binocular.

### 4.2. Transmission Electron Microscopy

Venom glands and 15,000× *g* MpVLP pellets were processed for transmission electron microscopy as described in [21]. Briefly, samples were fixed in sodium cacodylate (0.1 M, pH 7.2) for MpVLPs or in Insect Ringer for venom apparatus (KCl 182 mM; NaCl 46 mM; CaCl_2_ 3 mM; Tris-HCl 10 mM)) supplemented with 5% glutaraldehyde and stored for 24 h at 4 °C. Post-fixation was done with 2% osmium tetroxide in the same buffers, followed by dehydration in a graded ethanol series prior to inclusion in Epon and ultrathin section. The sections were contrasted with uranyl acetate and lead citrate before observation (1010, JEOL, Croissy, France and EM10CR, 80 kV, Zeiss, Marly le Roi, France).

### 4.3. Sequencing and Sequence Analyses

For transcriptomics, total RNA was extracted from 30 venom glands using TRIzol Reagent (Invitrogen, Courtaboeuf, France) according to the manufacturer’s instructions. After polyA selection, mRNA was fragmentated and the cDNA strand was synthesized using random primers. End repair, phosphorylation and A-tailing were done and, after ligation of adapters, a PCR amplification. Sequencing of PCR products was performed using Illumina RNA-Seq (HiSeq, 2 × 125 pb; Genewiz, Paris, France). The quality of the raw Illumina reads was controlled using FastQC software (mean quality score 36), and the reads were cleaned by removing low-quality sequences and reads containing N or adaptor sequences. *De novo* assembly was performed using CLC Genomics Server 8 (257,921,401 total reads; 206,153,609 matched reads; 177,571,432 reads in pairs; 14,926 contigs with 37% GC). The average length of the transcripts was 1604 bp and the N50 is 2153 bp. Raw transcriptomic reads of *M. pulchricornis* (PRJNA733444) and *L. javana* (PRJNA734452) are deposited at NCBI. The accession/description of the other species genomes and transcriptomes are in Table S2.

In order to calculate TPM values in venom glands and antennae, reads were aligned back to contigs with the Burrows-Wheeler Aligner (BWA-MEM, v 0.7.17) [93] with default parameters. Raw counts were calculated with the samtools idxstats (v1.10) [94]. TPM for a transcript (i) was calculated using the formula:

TPM_(I)_ = 1e^6^ × ((raw count_(i)_/transcript length_(i)_)/sum_(all dataset)_ (raw count/transcript length)) (1)

Peptides from transcriptomes of *M. pulchricornis*, *B. nigricans*, *L. javana*, *P. turionellae* and *P. concolor* were predicted with TransDecoder (v5.5; downloaded at https://github.com/brewsci/homebrew-bio/pkgs/container/bio%2Ftransdecoder, accessed on 17 July 2021), first by predicting the TransDecoder.LongOrfs ORFs with the -m 50 option, then by blasting the resulting longest ORFs (longest.orfs.pep) to the NCBI NR database (NR.2020-5-29), with DIAMOND (v2.0.6) [95] with the arguments: --max-target-seqs 1 --outfmt 6 --evalue 1e-5. Finally, we used the TransDecoder.Predict algorithm with the retain.blastp option with the DIAMOND results. Orthologs were identified by OrthoFinder (v2.3.8) [96] with the -S DIAMOND option and a database already built for previous analyses [97] and supplemented with the proteomes of *C. Congregata* [13], *A. ervi* and *L. fabarum* [98], and with the predicted peptides of *M. puchricornis*, *B. nigricans*, *L. javana*, *P. turionellae*, and *P. concolor* (see Table S2). Gene functions were obtained after a BLASTp of predicted peptides against NCBI NR (NR.2020-5-29) with DIAMOND (v2.0.6) and the arguments: --evalue 1e-8 --max-target-seqs 10 --masking 0, and InterProScan (v5.42-78) [99] with the options: -iprlookup -goterms --pathways -f xml -dp. Gene Ontology terms were assigned with Blast2GO CLI (v1.4.4) [100] using the results of Blastp and InterProScan.

### 4.4. Venom and VLPs Proteomic

Venom was obtained from the opening of 30 dissected reservoirs in a 50 µL drop in Insect Ringer supplemented with antiproteases (S8830; Sigma, Saint Quentin Fallavier, France) (IR + P). The extract was centrifuged at 500× *g* for 5 min. and the supernatant stored as total venom (TV). For MpVLPs purification, the supernatant centrifuged at 500× *g* was centrifuged again at 15,000× *g* for 10 min, the supernatant (Super) was removed and the pellet (Pellet) resuspended in 100 µL of IR + P and centrifuged again 10 min. at 15,000× *g*. The last pellet was resuspended in 50 µL of IR + P. All samples (TV, Super, and Pellet) were mixed with 4× Laemmli sample buffer with 10% β-mercaptoethanol [101]. Gel electrophoresis was carried out on a 12.5% acrylamide gel. After silver staining [102], gel slices were cut, washed with 50% acetonitrile, 50 mM NH_4_HCO_3_, and incubated overnight at 37 °C (with shaking) with 12.5 ng/mL trypsin (Promega, Charbonnières-les-Bains, France) in 25 mM NH_4_HCO_3_. The peptides were extracted three times with 50% acetonitrile, water containing 1% (*v*/*v*) formic acid and dried. Samples were analyzed online using a Q-orbitrap mass spectrometer (Q exactive, Thermo Fisher Scientific, Illkirch-Graffenstaden, France) coupled to an Ultimate 3000 HPLC (Dionex, Voisins-le-Bretonneux, France). Protein identification was performed using the Mascot v 2.3 algorithm (Matrix Science Inc., London, UK), by searching against the *M. pulchricornis* sequences. Peptides scoring higher than the identity score (*p* < 0.05) were considered significant. As the Mascot score for a protein is the summed score for the individual peptides, it can be used to estimate protein abundance. Such semiquantitative measure of protein abundance is called “spectral counting”, defined as the total number of spectra identified for a protein. Mass spectrometry proteomics raw data were deposited to the ProteomeXchange Consortium (http://proteomecentral.proteomexchange.org, 17 July 2021) via the MassIVE partner repository [103] with the dataset identifier PXD022771.

Mass spectrometry-matched sequences were manually verified and putative protein sequences blasted using BLASTp on NCBI NR for homologies (https://blast.ncbi.nlm.nih.gov/Blast.cgi, accessed on 17 July 2021) using default values. Protein domains were obtained from PFAM (http://pfam.xfam.orgresults, accessed during the 2020–2021 years) during NCBI blast and were also searched or confirmed using HMMERSCAN with default set up [104]. The presence of a N-terminus signal peptide sequence was tested using SignalP-5.0 server (http://www.cbs.dtu.dk/services/SignalP/, accessed during the 2020–2021 years) [105]. Sequence alignment was performed with MUSCLE (https://www.ebi.ac.uk/Tools/msa/muscle/, accessed on 17 July 2021) [106].

## Figures and Tables

**Table 1:** Sequencing coverage of predicted *M. pulchricornis* venom gland CDS (TPMv; number of sequences for given TPM (Transcripts Per Kilobase Million) ranges in venom gland transcriptome).

TPMv Range	Number of Predicted Proteins
75,000–10,000	15
9999–5000	24
4999–1000	88
999–500	118
499–100	480
99–50	477
49–25	852
24–10	2370
9–0	12,404

**Table 2:** List of the 39 CDS highly expressed in *M. pulchricornis* venom gland (TPMv > 5000)**.** CDS with TPMv/TPMa ratio > 500 are indicated in pink, in orange 500 > ratio > 200 and in green ratio < 50.

CDS ID	Sequence Description *	Species	TPMv	TPMa	TPMv/ TPMa	Orthogroup	*Mp*^§^ Proteins in the OG	Total Proteins in the OG
60.p3	BAL70305.1 hypothetical protein	*M. pulchricornis*	70,871	549	129			
64.p1	BAL70301.1 hypothetical protein	*M. pulchricornis*	31,382	115	272	OG0042547	1	2
32.p1	BAL70307.1 hypothetical protein	*M. pulchricornis*	30,408	124	245			
338.p1	BAL70305.1 hypothetical protein	*M. pulchricornis*	28,057	174	161	OG0042502	1	2
1043.p6	uncharacterized protein LOC115116642	*Oncorhynchus nerka*	18,777	111	169			
7952.p1	No hit		17,559	133	132			
38.p1	uncharacterized protein LOC103570348	*M. demolitor*	15,273	94	162	OG0042509	1	2
174.p2	BAL70304.1 hypothetical protein	*M. pulchricornis*	15,034	133	113	OG0019101	5	6
29.p1	BAL70308.1 hypothetical protein	*M. pulchricornis*	13,879	66	210	OG0042489	2	2
2896.p4	No hit		13,698	123	111	OG0042486	1	2
35.p1	No hit		13,271	131	101	OG0021629	4	5
4874.p12	No hit		12,545	103	121			
5.p1	BAL70306.1 hypothetical protein	*M. pulchricornis*	12,037	74	162	OG0019101	5	6
42.p1	No hit		10,282	56	183	OG0042514	1	2
57.p1	No hit		10,256	105	97	OG0042539	2	2
39.p1	BAL70290.1 hypothetical protein	*M. pulchricornis*	9755	71	137	OG0019101	5	6
121.p1	BAL70308.1 hypothetical protein	*M. pulchricornis*	9660	104	93	OG0025719	4	4
6996.p3	No hit		8834	57	155			
6621.p3	No hit		8315	73	114			
52.p1	BAL70302.1 hypothetical protein	*M. pulchricornis*	8230	13	633	OG0021663	4	5
281.p1	No hit		8078	17	475			
86.p1	No hit		8049	18	447	OG0025738	4	4
30.p1	No hit		7603	30	253	OG0042491	1	2
421.p1	Low quality protein, fibrillin-1- partial	*Anopheles darlingi*	7188	63	114	OG0009646	17	20
186.p3	No hit		6991	129	54			
72.p1	XP.023948444.1 uncharacterized protein LOC112053291 isoform X2	*Bicyclus anynana*	6968	36	193	OG0015773	7	8
73.p1	No hit		6695	28	239	OG0042561	1	2
21.p1	5NUC-like	*Apis cerana*	6656	4	1664	OG0031454	2	3
11189.p3	BAL70308.1 hypothetical protein	*M. pulchricornis*	6606	42	157	OG0025719	4	4
155.p1	No hit		5916	4	1479	OG0009638	18	20
2291.p1	PREDICTED: location of vulva defective 1 X1	*M. demolitor*	5903	145	40	OG0004499	3	36
4719.p2	No hit		5735	20	286			
285.p2	No hit		5626	29	194			
114.p1	Lipase-related 2	*Cephus cinctus*	5395	31	174	OG0010100	8	18
380.p3	No hit		5346	22	243			
3115.p10	No hit		5227	99	52			
49.p1	BAL70302.1 hypothetical protein	*M. pulchricornis*	5214	10	521			
932.p4	No hit		5076	436	11			
132.p1	No hit		5042	47	107	OG0015773	7	8

* best hit protein blast E < 10^−8^; ^§^, *Mp*: *M. pulchricornis.*

**Table 3:** Names and functions of the most abundant MpVLPs proteins. They are classified starting from the most abundant MpVLP protein using the Mascot “ranks” that ranged from 1 (the most abundant) to 516 (the less abundant) for MpVLPs and from 1 to 1354 for TV CDS/proteins, respectively.

CDS ° ID	Gel Bands *	Names and Functions of MpVLPs Proteins (E-Value Protein Blast) ^§^	MpVLPs Mascot Score (Rank)	TV Mascot Score (Rank)	TPMv/TPMa	OG
38	TV8-9 P4-5	Contig containing 2 CDS: - 38.p1, Uncharacterized protein LOC103577632 [*Microplitis demolitor*], 9 × 10^−14^. *DUF4803 domain,* 1.2 × 10^−13^. - 38.p2, Uncharacterized protein LOC106659714 [*Trichogramma pretiosum*], 5 × 10^−12^. *DUF4803 domain,* 7.2 × 10^−12^.	44,936 (1)	40,770 (1)	15,273/95 1309/11	OG0042509 OG0042510
35	TV10 P5	No hit	24,427 (2)	28,317 (2)	13,271/131	OG0021629
29	TV14-17 P6-8	Hypothetical protein [*Meteorus pulchricornis*] BAL70308.1, 3 × 10^−164^.	13,954 (3)	21,697 (4)	13,879/67	OG0042489
39	TV13-17 P8-9	Hypothetical protein [*Meteorus pulchricornis*] BAL70290.1., 7 × 10^−127^.	11,019 (4)	9659 (11)	9755/71	OG0019101
281	TV6-7 P2-3	No hit	9946 (5)	11,374 (10)	8078/17	na
72	TV7 P3	Uncharacterized protein LOC106655966 [*Trichogramma pretiosum*], 6 × 10^−11^. *DUF4803 domain,* 5.9 × 10^−10^.	9842 (6)	7277 (19)	6968/36	OG0015773
52	TV14-15 P7	Hypothetical protein [*Meteorus pulchricornis*] BAL70302.1, 1 × 10^−156^.	7157 (7)	12,900 (8)	8230/13	OG0021663
1173	TV21 P13	Hypothetical protein [*Meteorus pulchricornis*] BAL70304.1, 9 × 10^−42^.	6728 (8)	5156 (27)	4196/19	OG0019101
110	TV7 P3	Hypothetical protein [*Meteorus pulchricornis*] BAL70303.1, 2 × 10^−15^. *Metalloproteases (“zincins”), catalytic domain,* 9.2 × 10^−9^.	6535 (9)	3149 (41)	763/5	OG0000843
64	TV22-25 P12-13	Hypothetical protein [*Meteorus pulchricornis*] BAL70301.1, 3 × 10^−72^.	6517 (10)	15,886 (6)	31,382/115	OG0042547
42	TV14-15 P7	No hit	5465 (11)	6617 (21)	10,282/57	OG0042514
16	TV12-13 P6	A disintegrin and metalloproteinase with thrombospondin motifs 6-like [*Microplitis demolitor*], 1 × 10^−14^. *A disintegrin and metalloproteinase with thrombospondin motifs (Domain Metalloproteases (“zincins”), catalytic domain, Reprolysin-like*, 4.8 × 10^−9^.	5007 (12)	12,463 (9)	3528/17	OG0005678
6	TV18-19 P9-10	Contig containing 2 CDSs: - 6.p2, Uncharacterized protein LOC106128232 isoform X6 [*Papilio xuthus*], 6 × 10^−26^. *Growth factor receptor domain*, 4.5 × 10^−8^. - 6.p3, Fibrillin-1 [*Dufourea novaeangliae*], 7 × 10^−18^. *Putative Growth factor receptor domain,* 1.2 × 10^−5^.	4948 (13)	4063 (37)	2924/23 398/28	OG0009646 OG0009646
267	TV13-14 P6-7	Uncharacterized protein LOC105263636 [*Fopius arisanus*], 5 × 10^−8^. *Hemolymph juvenile hormone binding domain (JHBP domain),* 1.6 × 10^−11^.	4058 (14)	5138 (28)	2950/28	OG0025736
263	TV13-14 C7	Hypothetical protein [*Meteorus pulchricornis*] BAL70303.1., 4 × 10^−12^. *Metalloproteases (“zincins”), catalytic domain,* 1.7 × 10^−8^.	3397 (15)	4678 (33)	1340/6	OG0001872
109	TV13-14 P6-7	Hypothetical protein [*Meteorus pulchricornis*] BAL70303.1, 3 × 10^−8^.	2190 (16)	1630 (85)	522/1	OG0001872
73	TV23-24 P13	No hit	2131 (17)	7876 (16)	6695/28	OG0042561
285	TV22 P12	No hit	2076 (18)	1592 (73)	5625/28	na
230	TV5-6 P2	Unknown. *Lipocalins (Retinol binding protein-like) domain*, 5 × 10^−8^.	2071 (19)	2654 (48)	907/10	OG0031457
668	TV8-9 P4	Hypothetical protein [*Meteorus pulchricornis*] BAL70303.1, 4 × 10^−11^.	1812 (20)	2198 (57)	676/6	OG0000843
339	TV6 P2	Hypothetical protein [*Meteorus pulchricornis*] BAL70303.1, E = 0.0015.	1726 (21)	2811 (46)	544/5	OG0001872
126	TV11 & TV22 P12	A disintegrin and metalloproteinase with thrombospondin motifs 18-like [*Chelonus insularis*], 9 × 10^−16^. *Metalloproteases (“zincins”), catalytic domain,* 1.3 × 10^−8^.	1566 (22)	8290 (13)	1684/1	OG0000306
927	TV8 P3-4	Uncharacterized protein LOC118072154 [*Chelonus insularis*], 4 × 10^−15^. *Metalloproteases (“zincins”) catalytic domain,* 2 × 10^−9^.	1531 (23)	2074 (61)	285/23	OG0000306
5	TV20-21 P11	Hypothetical protein [*Meteorus pulchricornis*] BAL70306.1, 1 × 10^−92^.	1511 (24)	7780 (17)	12,037/74	OG0019101
8	TV18-19 P10-11	No hit	1486 (25)	5084 (29)	1622/7	OG0031468
1266	TV6 P2	No hit	1476 (26)	1598 (72)	285/1	OG0000306
1636	TV7 P3	No hit	1463 (27)	1578 (88)	235/0.3	OG0000843
875	TV9 P4	No hit	1376 (28)	1296 (78)	1114/8	OG0031455
208	TV12-13 P6	Hypothetical protein [*Meteorus pulchricornis*] BAL70303.1, 2 × 10^−14^. *Collagenase catalytic domain,* 3.3 × 10^−5^.	1368 (29)	1575 (68)	1051/5	OG0001872
1290	TV15 P7-8	Uncharacterized protein LOC118072154 [*Chelonus insularis*], 4 × 10^−19^. *Metalloproteases (“zincins”), catalytic domain,* 6.1 × 10^−12^.	1356 (30)	2543 (57)	188/3	OG0005678
1198	TV7 P3	Hypothetical protein [*Meteorus pulchricornis*] BAL70303.1, 4 × 10^−13^.	1276 (31)	635 (146)	948/6	OG0000306
2114	P3-4	No hit	1259 (32)	918 (108)	480/3	OG0000306
1039	P2	Fibrillin-2 like protein [*Argiope bruennichi*], 8 × 10^−24^.	1086 (33)	800 (125)	386/2	OG0009646
379.p2	P3	Hypothetical protein [*Meteorus pulchricornis*] BAL70303.1, 6 × 10^−17^.	1025 (34)	1057 (94)	429/7	OG0000843
647	TV7 P4	Hypothetical protein [*Meteorus pulchricornis*] BAL70303.1, 4 × 10^−14^. *Metalloproteases (“zincins”), catalytic domain,* 2 × 10^−6^.	1023 (35)	1282 (80)	242/2	OG0000843
897	TV3-4 P1	Venom protein 2 [*Microctonus hyperodae*], 7 × 10^−122^. *DUF 4803 domain,* 3.4 × 10^−23^.	952 (36)	21,507 (5)	262/7	OG0007440
293	TV8 P3-4	Hyaluronidase [*Diachasma alloeum*], 6 × 10^−67^. *Hyaluronidase (Hyaluronidase domain),* 5.2 × 10^−60^.	934 (37)	902 (110)	363/1	OG0005785
686	P2	No hit	930 (38)	956 (104)	342/2	OG0042554
496	P6	Venom metalloproteinase 2-like [*Microplitis demolitor*], 8 × 10^−15^. *Collagenase (Catalytic Domain),* 2.5 × 10^−14^.	921 (39)	1168 (89)	279/4	OG0009638
91	TV6 P9	Venom metalloproteinase 3-like [*Pogonomyrmex barbatus*], 3 × 10^−17^. *Metallo-peptidase family M12 domain,* 2.7 × 10^−6^.	915 (40)	1185 (110)	182/1	OG0000306
575	P3	Hypothetical protein [*Meteorus pulchricornis*] BAL70303.1, E = 0.001.	910 (41)	839 (153)	952/6	OG0000843
590	P3	Unknown. *Putative Serpin domain,* 2.8 × 10^−6^.	896 (42)	483 (223)	641/4	OG0042523
153	TV13 P6	Hypothetical protein [*Meteorus pulchricornis*] BAL70303.1, 9 × 10^−14^. *ADAM cysteine-rich domain,* 2.4 × 10^−5^.	818 (43)	2230 (56)	1637/11	OG0001872
651	TV10-11 P5 & P9-10	Venom metalloproteinase 3-like [*Chelonus insularis*], 1 × 10^−6^. *Collagenase Catalytic Domain,* 1.2 × 10^−6^.	804 (44)	2022 (63)	2059/4	OG0009638
2103	P4	Uncharacterized protein LOC118072154 [*Chelonus insularis*], 8 × 10^−12^.	801 (45)	1171 (112)	100/3	OG0000843
4685	TV10 P5	No hit	766 (46)	881 (114)	4416/30	na
375	TV5 P2	Venom metalloproteinase 2-like [*Microplitis demolitor*], 3 × 10^−10^. *Metalloproteases (“zincins”), catalytic domain,* 3.7 × 10^−9^.	760 (47)	1638 (71)	161/4	OG0000843
216.p1	TV7	Uncharacterized protein LOC118072154 [*Chelonus insularis*], 1 × 10^−7^. *Collagenase catalytic domain,* 1.1 × 10^−5^.	710 (48)	1227 (84)	959/7	OG0000306
609	P2	Unknown. *Calycin beta-barrel core domain,* 8.1 × 10^−5^.	697 (49)	601 (153)	238/4	OG0021629
429.p1	TV10-11 & TV23 P5-P13	Uncharacterized protein LOC118072154 [*Chelonus insularis*], 2 × 10^−20^. *Metalloproteases (“zincins”), catalytic domain,* 2.8 × 10^−10^.	691 (50)	3131 (42)	439/11	OG0000306
286	TV14 & TV22 P12	Hypothetical protein [*Meteorus pulchricornis*] BAL70303.1, 2 × 10^−9^. *Metalloproteases (“zincins”), catalytic domain,* 1.6 × 10^−8^.	690 (51)	1198 (107)	506/1	OG0001872
244	TV11-12 P8	A disintegrin and metalloproteinase with thrombospondin motifs 15-like [*Trichogramma pretiosum*], 7 × 10^−14^. *Metalloproteases (“zincins”), catalytic domain,* 4.1 × 10^−11^.	699 (52)	3022 (44)	501/4	OG0009638
554	P5	Pancreatic lipase-related protein 2-like [*Temnothorax curvispinosus*], 3 × 10^−68^. *Pancreatic lipase-like enzymes domain,* 9.5 × 10^−91^.	611 (53)	1258 (82)	602/9	OG0010100
124	TV20-21 P11	Hypothetical predicted protein [*Cloeon dipterum*], 9 × 10^−8^.	594 (54)	1018 (97)	1243/6	OG0019104
185	TV12-13	Hypothetical protein [*Meteorus pulchricornis*] BAL70303.1, 5 × 10^−8^. *Metalloproteases (“zincins”), catalytic domain,* 4.6 × 10^−7^.	594 (55)	2979 (50)	789/3	OG0001872
1150	-	No hit. *Lipocalins superfamily (Retinol binding protein-like),* 2.1 × 10^−6^.	584 (56)	530 (164)	113/1	OG0021629
166	-	No hit	549 (57)	438 (181)	557/5	OG0000306
378.p2	-	A disintegrin and metalloproteinase with thrombospondin motifs 1-like [*Microplitis demolitor*], 3 × 10^−8^. *ADAM cysteine-rich domain,* 3.4 × 10^−5^.	532 (58)	204 (286)	359/6	OG0000306
943	-	No hit	530 (59)	556 (157)	101/2	OG0000306
1411	P6	Hypothetical protein [*Meteorus pulchricornis*] BAL70303.1, 9 × 10^−15^.	523 (60)	738 (136)	238/0.3	OG0001872

° contigs are .p1 unless indicated; * presence in TV and Pellet (P) bands from Figure 3 are indicated when the protein was within the 15 highest validated mascot scores in the bands (a protein may have a high total mascot score, but not be among the most abundant in a single band). ^§^, In italic, the domain search results.

**Table 4:** Names and functions of the most abundant venom soluble proteins. Mascot “ranks” range from 1 (the most abundant) to 1354 (the less abundant) for TV CDS/proteins and from 1 to 516 for MpVLP CDS/proteins.

CDS °	Gel Bands *	Names and Functions of Venom Soluble Proteins (E-Value Protein Blast) ^§^	TV Score (Rank)	MpVLPs Score (Rank)	TPMv/TPMa	Contig OG
10764	TV9-12	Uncharacterized protein LOC103570348 [*Microplitis demolitor*], 6 × 10^−22^. *DUF4803 domain,* 5.2 × 10^−16^.	23,572 (3)	16 (489)	0.9/0.3	OG0015773
536	TV12-13	No hit. *Lipocalins (Retinol binding protein-like) domain,* 8.5 × 10^−10^.	14,497 (7)	278 (87)	581/30	OG0015781
341	TV3-4	Venom protein 2 [*Microctonus hyperodae*] 2 × 10^−58^. *DUF4803 domain,* 3.2 × 10^−14^.	9650 (12)	429 (69)	196/4	OG0025733
21	TV4-5	Protein 5NUC [*Apis mellifera*], 9 × 10^−53^. *5’-nucleotidase domain,* 1.5 × 10^−32^.	8248 (14)	125 (136)	6656/4	OG0002704
114	TV11-13	Pancreatic lipase-related protein 2 [*Bombus vancouverensis nearcticus*], 5 × 10^−52^. *Pancreatic lipase N-terminus domain,* 2.6 × 10^−48^.	7962 (15)	106 (151)	5394/31	OG0010100
8250	TV3-4	Uncharacterized protein LOC105686340 isoform X1 [*Athalia rosae*], 3 × 10^−59^. *DUF4803 domain,* 3.7 × 10^−11^.	7489 (18)	60 (214)	1/4	OG0010765
7504	TV4	Uncharacterized protein LOC107042423 isoform X1 [*Diachasma alloeum*], 3 × 10^−53^. *DUF4803 domain,* 2.4 × 10^−25^.	6704 (20)	173 (118)	0.4/5	OG0010765
2792	TV10	Uncharacterized protein LOC103574503 [*Microplitis demolitor*], 1 × 10^−28^. *DUF4803 domain,* 4.9 × 10^−8^.	6520 (22)	73 (183)	5/6	OG0005090
136	TV13	No hit. *Collagenase (Catalytic Domain),* 1.7 × 10^−9^.	6406 (23)	193 (104)	4945/25	OG0000843
106	TV5-6	Predicted pancreatic triacylglycerol lipase [*Wasmannia auropunctata*], 9 × 10^−35^. *Pancreatic lipase N-terminus domain,* 4.2 × 10^−41^.	6267 (24)	63 (209)	3310/21	OG0031414
2331	TV19-21	No hit. *Calycin beta-barrel core domain,* 6.3 × 10^−8^.	5752 (25)	175 (114)	52/8	OG0015781
3422	TV10-11	Arginine kinase [*Microplitis demolitor*], E = 0.0. *Phosphagen (guanidino) kinases such as arginine kinase and similar enzymes domain,* E = 0.0.	5653 (26)	49 (238)	1/764	OG0001469
58	TV11-12	A disintegrin and metalloproteinase with thrombospondin motifs 6 [*Galendromus occidentalis*], 5 × 10^−12^. *Metalloproteases (“zincins”), catalytic domain,* 1.1 × 10^−7^.	5079 (30)	444 (66)	1579/8	OG0009638
8023	TV4	Venom protein [*Ampulex compressa*], 8 × 10^−56^. *Two DUF4803 domains,* 4.9 × 10^−14^ and 6.5 × 10^−10^.	4972 (31)	276 (88)	3/2	OG0010765
11420	TV4-5	Uncharacterized protein LOC115884266 [*Sitophilus oryzae*], 3 × 10^−37^. *DUF4803 domain*, 3.9 × 10^−^^16^.	4758 (32)	118 (141)	298/6	OG0010765
11339	TV9-10, TV20	γ-Glutamyltranspeptidase 1 isoform X1 [*Microplitis demolitor*] E = 0.0. *γ-glutamyltranspeptidase domain,* 2.4 × 10^−156^.	4507 (34)	101 (122)	0.8 /3	OG0000441
662.p2	TV19	Hypothetical protein [*Meteorus pulchricornis*] BAL70297.1, 5 × 10^−139^.	4465 (35)	181 (111)	1321/49	OG0042432
1164	TV3	Venom protein 2 [*Microctonus hyperodae*], 2 × 10^−54^.	4306 (36)	not found	11/6	OG0008161
2455	TV3	Hypothetical protein TSAR.007430 [*Trichomalopsis sarcophagae*], 2 × 10^−85^. *Eukaryotic CAP domain protein (cysteine-rich secretory proteins, antigen 5, and pathogenesis-related 1 proteins),* 7.1 × 10^−110^.	3922 (38)	not found	32/1	OG0000373
11685	TV4	Uncharacterized protein LOC105686340 isoform X2 [*Athalia rosae*], 1 × 10^−17^. *DUF4803 domain,* 5.7 × 10^−25^.	3866 (39)	118 (142)	0.4/2	OG0010765
275	TV4	Uncharacterized protein LOC108086051 [*Drosophila kikkawai*], 2 × 10^−13^. *DUF4803 domain,* 1.4 × 10^−22^.	3455 (40)	65 (195)	3317 /19	OG0025743
6649	TV1	Myosin heavy chain, muscle isoform X5 [*Microplitis demolitor*], E = 0.0. *Class II myosin heavy chain 1, motor domain,* E = 0.0.	3032 (43)	not found	0.5/153	OG0000051
69	TV13	Glyceraldehyde-3-phosphate dehydrogenase [*Athalia rosae*], E = 0.0. *Glyceraldehyde-3-phosphate dehydrogenase domain,* E = 0.0.	2911 (45)	not found	205/1324	OG0003321
229	TV22	No match	2693 (47)	109 (147)	4413/13	OG0001872
726	TV3	Uncharacterized protein LOC118068317 [*Chelonus insularis*], 2 × 10^−52^. *DUF4803 domain,* 9.3 × 10^−36^.	2551 (49)	not found	197/2	OG0019097
53	TV4	Receptor-type tyrosine-protein phosphatase epsilon isoform X2 [*Tribolium castaneum*], 7 × 10^−16^.	2514 (51)	190 (107)	4243/31	OG0010765
155	TV11	Hypothetical protein [*Meteorus pulchricornis*] BAL70303.1, 7 × 10^−6^. *Collagenase domain,* 2.1 × 10^−7^.	2458 (52)	not found	5915 /4	OG0009638
4840	TV1	Apolipophorins [*Microplitis demolitor*], 4 × 10^−48^.	2451 (53)	51 (232)	0.4/189	OG0013776
763	TV12	Predicted aldose reductase [*Microplitis demolitor*], E = 0.0. *AKR1A family of aldo-keto reductase (AKR),* 1.2 × 10^−167^.	2365 (54)	not found	112/257	OG0000155
14605	TV1	Apolipophorins [*Chelonus insularis*], 2 × 10^−48^.	2303 (55)	not found	0.1/17	OG0025456
1036	TV16	Putative 14-3-3 protein zeta isoform X1, partial [*Cotesia chilonis*], 4 × 10^−172^. *14-3-3 protein domain,* 48 × 10^−166^.	2188 (58)	148 (126)	309/970	OG0000955
11280	TV25	Odorant-binding protein 3 [*Meteorus pulchricornis*], 1 × 10^−92^. *Domain PBP/GOBP family,* 1 × 10^−22^.	2175 (59)	not found	2/3700	OG0000832
6971	TV3	Neprilysin-2-like isoform X2 [*Belonocnema treatae*], 6 × 10^−67^. *Peptidase family M13 includes neprilysin, endothelin-converting enzyme I,* 1.98 × 10^−73^.	2098 (60)	not found	1/1	OG0017160

° contigs are .p1 unless indicated; *, Band number from Figure 3; ^§^, in italic the domain search results.

## Data Availability

Raw transcriptomic reads of *M. pulchricornis* (PRJNA733444) and *L. javana* (PRJNA734452) are deposited at NCBI. Mass spectrometry proteomics raw data were deposited to the ProteomeXchange Consortium (http://proteomecentral.proteomexchange.org accessed on 17 July 2021) via the MassIVE partner repository with the dataset identifier PXD022771.

## Supplementary Materials

The following are available online at https://www.mdpi.com/article/10.3390/toxins13070502/s1, Figure S1: Phylogeny from Orthofinder and Blast2GO Short Reports (pdf file), Figure S2: Comparison of the CDSs encoded by contig 38 (pdf file), Figure S3: Sequence comparison of the venom metalloproteases (pdf file), Figure S4: Sequence comparison of the DUF4803 containing proteins (pdf file), Table S1: General analysis of the *M. pulchricornis* gland transcriptome (excel file), Table S2: Genomes and transcriptomes used in this study (pdf file), Table S3: Data and sequence of the venom metalloproteases (excel file), Table S4: Data and sequence of the venom DUF4803 containing proteins (excel file).
