# Supplementary material for: Proteo-Trancriptomic Analyses Reveal a Large Expansion of Metalloprotease-Like Proteins in Atypical Venom Vesicles of the Wasp Meteorus pulchricornis (Braconidae)
Source: Toxins (Basel). 2021 Jul 19;13(7):502. doi: 10.3390/toxins13070502 (PMC8310156; doi:10.3390/toxins13070502)
Supplement: Supplementary file 1 [file toxins-13-00502-s001.zip › 2021 toxins-supplementary-1282739.pdf]

Supplementary Materials: Proteo-Trancriptomic Analyses Reveal a Large Expansion of Metalloprotease-Like Proteins in Atypical Venom Vesicles of the Wasp *Meteorus pulchricornis* (Braconidae)

Jean-Luc Gatti, Maya Belghazi, Fabrice Legeai, Marc Ravallec, Marie Frayssinet, Stéphanie Robin, Djibril Aboubakar-Souna, Ramasamy Srinivasan, Manuele Tamò, Marylène Poirié and Anne-Nathalie Volkoff

For Table S1, S3, S4, see excel files

Table S2. Genomes and Transcriptomes use in the study

	Species	Version	Number of CDS	Site	Reference (DOI)	NCBI Project
Genomes	*Aphidius ervi*	3.0	20,344	BIPAA	10.1186/s12864-020-6764-0	PRJNA347567
	Apis mellifera	HAv3.1	23,471	NCBI	10.1186/s12864-019-5642-0	PRJNA471592
	Campoletis sonorensis	1.1	21,987	BIPAA	10.1186/s12915-020-00822-3	PRJNA590982
	Cotesia congregata	2.3	14,149	BIPAA	10.1038/s42003-020-01623-8	
	Diachasma alloeum	GCF_001412515.1	19,692	NCBI	10.1093/gbe/evz205.	PRJNA306876
	Drosophila melanogaster	r6.26	30,480	FLYBASE	10.1101/gr.185579.114	
	Fopius arisanus	ASM80636v1	18,906	NCBI	10.1534/g3.117.040741	PRJNA258104
	Hyposoter didymator	2.1	18,154	BIPAA	10.1186/s12915-020-00822-3	PRJNA589497
	Lysiphlebus fabarum	1.0	15,203	BIPAA	10.1186/s12864-020-6764-0	PRJNA587428
	Microplitis demolitor	GCF_000572035.2	18,586	NCBI	10.1534/g3.118.200308	PRJNA251518
	Nasonia vitripennis	GCF_000002325.3	24,846	NCBI	10.1126/science.1178028	PRJNA13660
	Venturia canescens	1.1	23,401	BIPAA	10.1126/sciadv.1501150	

**Transcriptomes of venom glands**	***Bracon nigricans***	**1.0**	**57,686**		**10.1186/s12864-019-6396-4**	**PRJNA542369**
	Liragathis javana	**1.0**	**11,337**	**BIPAA**		**PRJNA734452**
	Meteorus pulchricornis	**1.0**	**16,828**	**BIPAA**		**PRJNA733444**
	Pimpla turionnalae	**1.0**	**347,311**	**https://zenodo.org/record/3545834#.YBgwJ3VKiV4**	**10.1186/s12864-019-6396-4**	**PRJNA555750**
	Psyttalia concolor	**1.0**	**14,370**		**10.1038/srep35873**	**PRJNA262710**
**Transcri-ptome of antennae**	***Meteorus pulchiricronis***				**10.1016/j.cbd.2017.01.002**	**PRJNA345399**

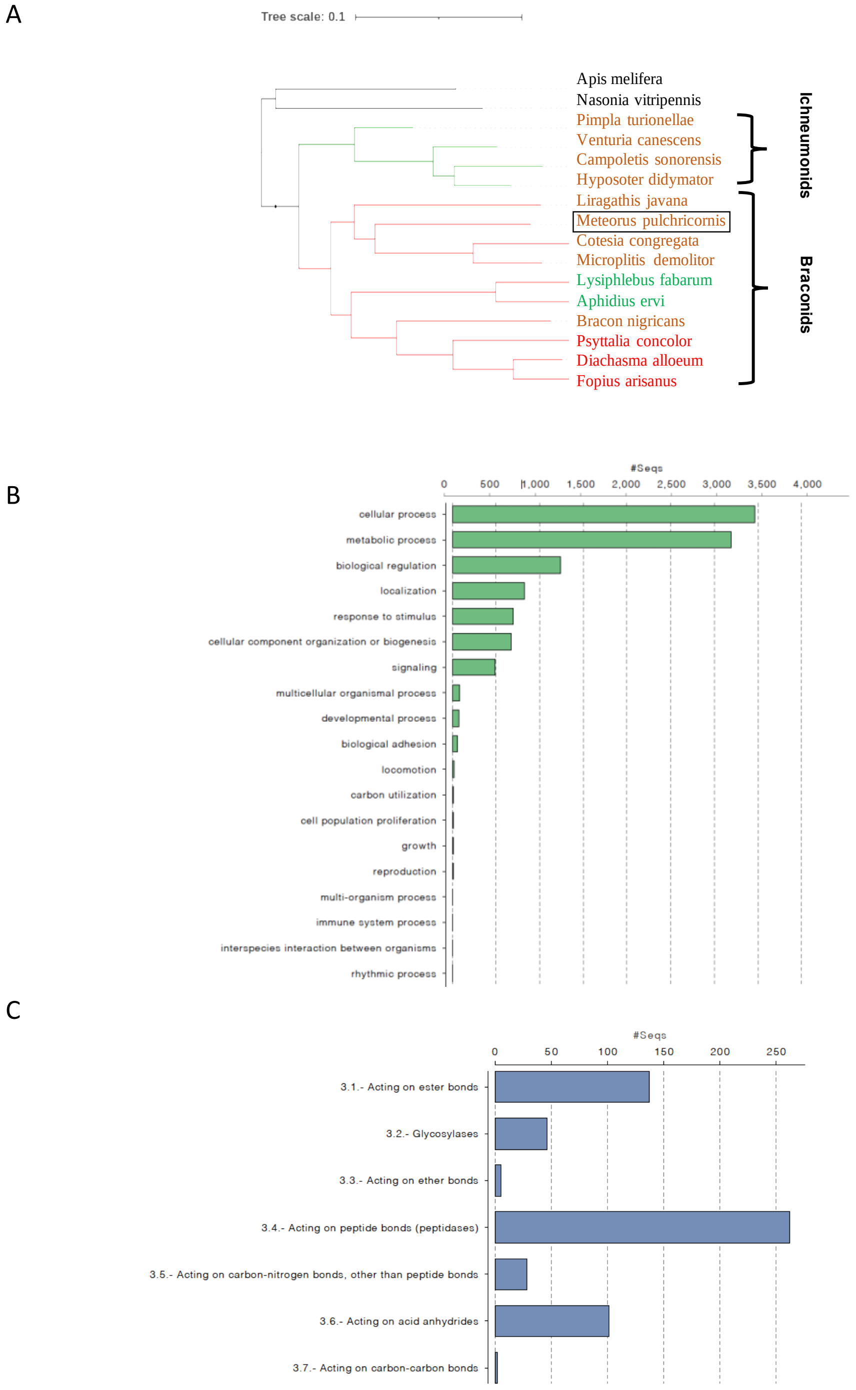

Figure S1. Phylogeny from Orthofinder and Blast2GO Short Reports. In A : phylogeny of the species from orthofinder (names: in black, non-Ichneumonidae; in orange, parasitoids of lepidopterans; in green, of aphids; in red, of dipterans). B, Second level GO categories of biological process and molecular function among functionally annotated *M. pulchricornis* transcripts. In C: Enzyme Code Level 3 Distribution.

```
MGPIIINFVV TFIMMYCEGI AGEVINNDTI NLEYFQHPFF FNMVHAKHQC
RKSCEDITMD DMTIQTDSDI KSSCYGTYND CRAMVVADTV DLQYCRKNNS
TDIVWAFDSQ RKVEYGQKSN CGDGATEYTV PRVGYSGGSG CPICVCTCYE
ATSGMASNKG KWWFSAVPQT SDENKVITGV KFVEINHTIF LQIKQNQFPI
DTTGNWKTLD EEVEAEEGVS KDTRLLLTDK RRSLMLDEFE LPDGYVITGV
KFEEATIPTT SLLAIRLSVR STKLTKSKDG LESETSTWIH GTTSKLKSTH
LKWLPSSMGL NPDLKTHIMF GIGDLSKDFG KLTEPTISAE PCEATILKET
PMGGLQLLLV NISEKQKIYP RVAPLGIDPD PESKEEEDDD DDMGDNGDSA
STTNLQFILI ALVPLSIIFN LFD
```

```
MGPIIINFVV TFIMMYCEGI AGEVINNDTI NLEYFQHPFF FNMVHAKHQC
RKSCEDITMD DMTIQTDSDI KSSCYGTYND CRAMVVADTV DLQYCRKNNS
TDIVWAFDSQ RKVEYGQKSN CGDGATEYTV PRVGYSGGSG CPICVCTCYE
ATSGMASNKG KWWFSAVPQT SDENKVITGV KFVEINHTIF LQIKQNQFPI
DTTGNWKTLD EEVEAEEGVS KDTRLLLTDK RRSLMLDEFE LPDGYVITGV
KFEEATIPTT SLLAIRLSVR STKLTKSKDG LESETSTWIH GTTSKLKSTH
LKWLPSSMGL NPDLKTHIMF GIGDLSKDFG KLTEPTISAE PCEATILKET
PMGGLQLLLV NISEKQKIYP RVAPLGIDPD PESKEEEDDD DDMGDNGDSA
STTNLQFILI ALVPLSIIFN LFD
```

```
MEKLSMNLVV ILMIIHCKEI AGDLINLKYF REAIIFSEHD LGTCGFGCGK
LTLSETANYK STFTKQVCDG YLVNCQTIVG KANASLQFCL DDQNSRMVWA
ENKQYGGAQY GIQPRECITP INEPLKTKDS ETCEACVCTC YHERESSEDE
NVWRFSLAEE TSGDAKVVTG VRFTLIDNII HLQIKENDLI STTNGTWKSI
TVDSDRDGPD TRKYATLSHN LQTIKLNQIF SPENSVITGV KFVKVPNLDK
KGFALELQIR STKLNDENDA LDPRHQDWHS GDPSTLTAGP RWWPDRGIHN
TPLPIVSLNR TSLNKDFGMT TNPQFYADPV VSCAQHDYPL KGIQLLLVEL
GGQEVIIPRI SPAIIKGSTL PDYLDYESNS EPELTDSANT ANLQFCLIAM
LPLLTIWNLF N
```

```
MEKLSMNLVV ILMIIHCKEI AGDLINLKYF REAIIFSEHD LGTCGFGCGK
LTLSETANYK STFTKQVCDG YLVNCQTIVG KANASLQFCL DDQNSRMVWA
ENKQYGGAQY GIQPRECITP INEPLKTKDS ETCEACVCTC YHERESSEDE
NVWRFSLAEE TSGDAKVVTG VRFTLIDNII HLQIKENDLI STTNGTWKSI
TVDSDRDGPD TRKYATLSHN LQTIKLNQIF SPENSVITGV KFVKVPNLDK
KGFALELQIR STKLNDENDA LDPRHQDWHS GDPSTLTAGP RWWPDRGIHN
TPLPIVSLNR TSLNKDFGMT TNPQFYADPV VSCAQHDYPL KGIQLLLVEL
GGQEVIIPRI SPAIIKGSTL PDYLDYESNS EPELTDSANT ANLQFCLIAM
LPLLTIWNLF N
```

Score	Expect	Method	Identities	Positives	Gaps
190 bits(482)	2e-60	Compositional matrix adjust.	133/427(31%)	207/427(48%)	20/427(4%)

```
Query  1    MGPIIINFVVTFIMMYCEGIAGEVINNDTINLEYFQHPFFFNMVHAKHQCRKSCEDITMD  60
            M   + +N VV   ++++C+ IAG     D INL+YF+     F+  H    C   C  +T+
Sbjct  1    MEKLSMNLVVILMIIHCKEIAG-----DLINLKYFREAIIFSE-HDLGTCGFGCGKLTLS  54

Query  61   DMTIQTDSDIKSSCYGTYNDCRAMVVADTVDLQYCRKNNSTDIVWAFDSQRK-VEYG-QK  118
            +       +  K  C G   +C+ +V        LQ+C  + ++ +VWA + Q     +YG Q
Sbjct  55   ETANYKSTFTKQVCDGYLVNCQTIVGKANASLQFCLDDQNSRMVWAENKQYGGAQYGIQP  114

Query  119  SNCGDGATEYTVPRVGYSGGSGCPICVCTCYEATSGMASNKGKWWFSAVPQTSDENKVIT  178
              C     E     +        C  CVCTCY        + ++  W FS   +TS + KV+T
Sbjct  115  RECITPINE----PLKTKDSETCEACVCTCYHERES-SEDENVWRFSLAEETSGDAKVVT  169

Query  179  GVKFVEINHTIFLQIKQNQFPIDTTGNWKTLDEEVEAEEGVSKDTR--LLLTDKRRSLML  236
            GV+F  I++ I LQIK+N      T G WK++  + + +       DTR    L+    +++ L
Sbjct  170  GVRFTLIDNIIHLQIKENDLISTTNGTWKSITVDSDRD---GPDTRKYATLSHNLQTIKL  226

Query  237  DEFELPDGYVITGVKFEEATIPTTSLLAIRLSVRSTKLTKSKDGLESETSTWIHGTTSKL  296
            ++    P+  VITGVKF  +          A+ L +RSTKL     D L+      W  G  S L
Sbjct  227  NQIFSPENSVITGVKFVKVPNLDKKGFALELQIRSTKLNDENDALDPRHQDWHSGDPSTL  286

Query  297  KSTHLKWLPSSMGLNPDLKTHIMFGIGDLSKDFGKLTEPTISAEPCEATILKETPMGGLQ  356
             +    +W P       N  L    +       L+KDFG  T P    A+P  +      + P+ G+Q
Sbjct  287  TAGP-RWWPDRGIHNTPLPI-VSLNRTSLNKDFGMTTNPQFYADPVVSCAQHDYPLKGIQ  344

Query  357  LLLVNISEKQKIYPRVAPLGIDPDPESKEEEDDDDDMGDNGDSASTTNLQFILIALVPLS  416
            LLLV +   ++ I PR++P   I                + + +    +  DSA+T NLQF LIA++PL
Sbjct  345  LLLVELGGQEVIIPRISPAIIKGSTLPDYLDYESNSEPELTDSANTANLQFCLIAMLPLL  404

Query  417  IIFNLFD  423
             I+NLF+
Sbjct  405  TIWNLFN  411
```

Figure S2. Comparison of Contig 38.p1 and .p2. Highlight in yellow putative signal peptide. In red peptides found by mass spectrometry in the total venom or the MpVLPs samples (in brown those found only in TV). The lower figure shows the Blastp comparison of the two proteins.

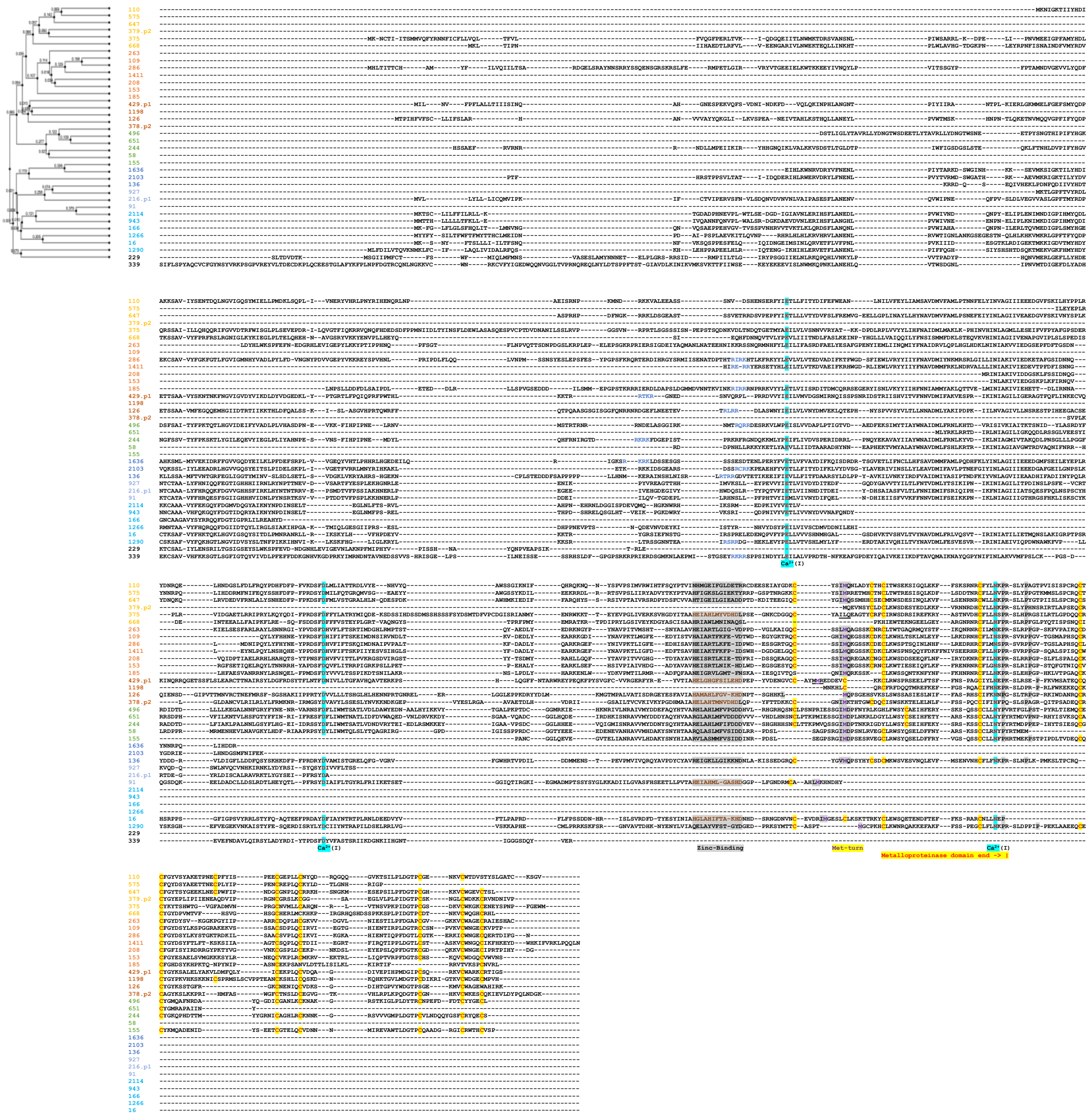

Figure S3. CLUSTAL multiple sequence alignment of the *M. pulchricornis* venom Metalloproneinases. Proteins with name of the same color clustered together and may form a clade as shown by the clustal clustering on the left. AA in blue, putative Furin site; AA in red highlight in cyan, putative triad involved in Ca2+ binding; Conserved Cys in red highlight in yellow; Highlight in grey sequence

features, AA in brown canonic Zn^{2+} binding/active site and in purple Met-turn.

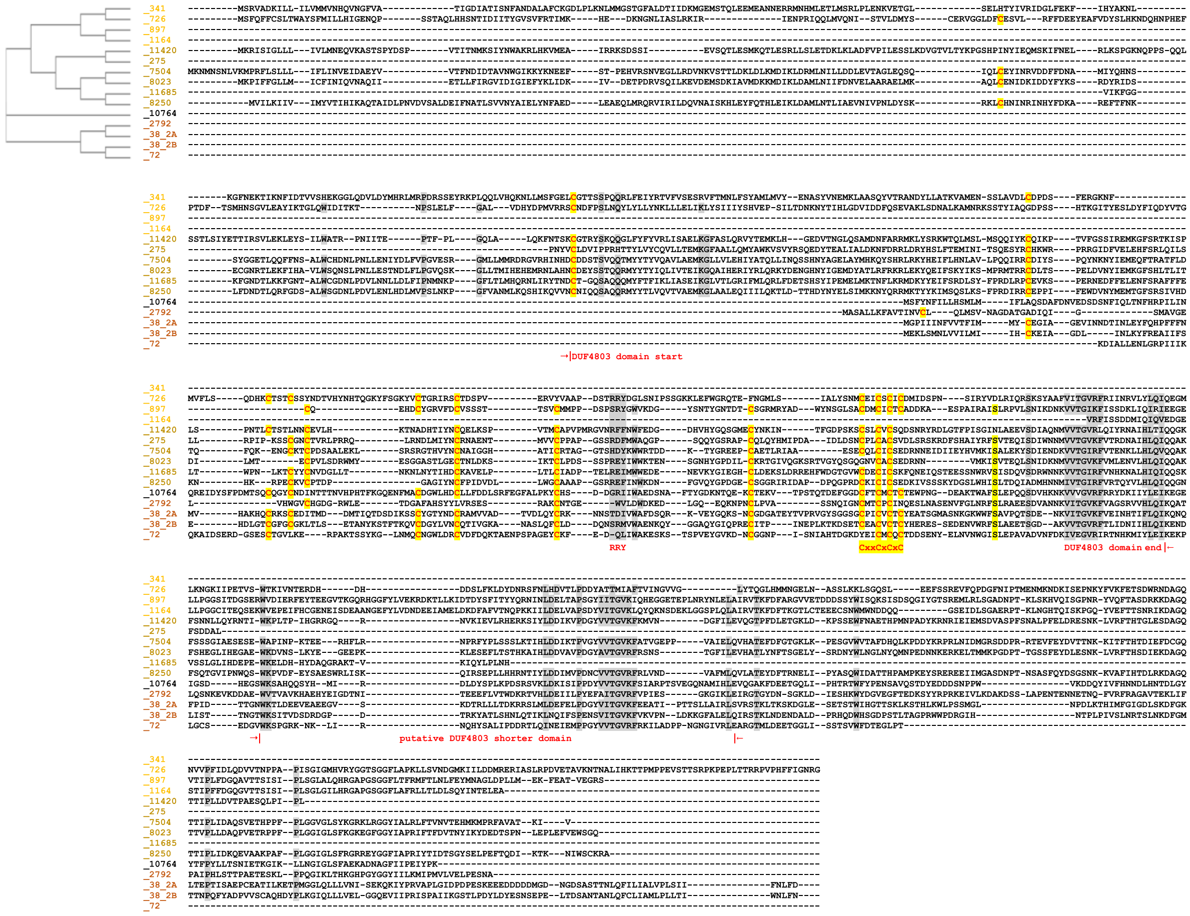

Figure S4. CLUSTAL multiple sequence alignment of the DUF4803 containing proteins. Proteins with name of the same color clustered together and may form a clade as shown by the clustal clustering on the left. Conserved Cys in red highlight in yellow; Highlight in grey sequence features and highly conserved AA.
